# P–C, P–N, and M–N Bond Formation Processes in Reactions of Heterometallic Phosphinidene-Bridged MoMn and MoRe Complexes with Diazoalkanes and Organic Azides to Build Three- to Five-Membered Phosphametallacycles

**DOI:** 10.1021/acs.inorgchem.2c02720

**Published:** 2022-11-09

**Authors:** M. Angeles Alvarez, Pablo M. Cuervo, M. Esther García, Miguel A. Ruiz, Patricia Vega

**Affiliations:** Departamento de Química Orgánica e Inorgánica/IUQOEM, Universidad de Oviedo, E-33071 Oviedo, Spain

## Abstract

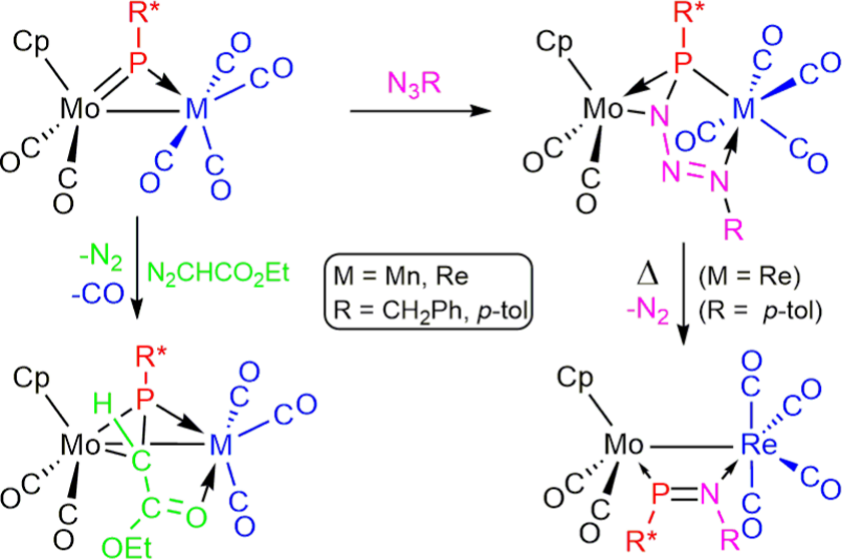

Reactions of the heterometallic MoRe complex [MoReCp(μ-PR*)(CO)_6_] and its MoMn analogue with some small molecules having N–N multiple bonds, such as diazoalkanes and organic azides, were investigated (R* = 2,4,6-C_6_H_2_^*t*^Bu_3_). Reactions with excess ethyl diazoacetate proceeded slowly and with concomitant denitrogenation to give complexes [MoMCp(μ-η^2^_P,C_:κ^2^_P,O_-PR*CHCO_2_Et)(CO)_5_], which display a bridging phosphaalkene ligand in a novel μ-η^2^:κ^2^ coordination mode, while reactions with other diazoalkanes resulted only in the decomposition of the organic reagent. The MoRe complex reacted with benzyl- or *p*-tolyl azide at room temperature to give the green complexes [MoReCp(μ-η^2^_P,N_:κ_P,N′_^2^-PR*N_3_R)(CO)_6_] [R = Bn, *p*-tol], which display bridging phosphatriazadiene ligands in a novel 6-electron donor coordination mode as a result of a formal [2 + 1] cycloaddition of the terminal N atom of the azide to the Mo–P double bond of the parent complex, followed by coordination of the distal NR nitrogen to the rhenium center. Denitrogenation was only observed for the *p*-tolyl azide derivative, which upon heating at 333 K yielded [MoReCp{μ-κ_P_:κ_N_-PR*N(*p*-tol)}(CO)_6_], a molecule displaying a bridging phosphaimine ligand in a rare κ_P_:κ_N_ coordination mode. Analogous reactions of the MoMn phosphinidene complex proceeded similarly at 273 K to give the phosphatriazadiene-bridged derivatives [MoMnCp(μ-η^2^_P,N_:κ^2^_P,N′_-PR*N_3_R)(CO)_6_], but these were thermally unstable and degraded at room temperature to give the mononuclear triazenylphosphanyl complexes [Mn^2^(κ_P,N_-PR*NHNNR)(CO)_3_] as major products, along with small amounts of the phosphaimine-bridged complex [MoMnCp{μ-κ_P_:κ_N_-PR*N(*p*-tol)}(CO)_6_] in the case of the *p*-tolyl azide derivative. The structure of the new complexes was analyzed in light of spectroscopic data and single-crystal diffraction studies on selected examples of each type of complex.

## Abstract 2

Reactions of [MoMCp(μ-PR)(CO)_6_] (M = Mn, Re) with N_2_CHCO_2_Et involved denitrogenation and decarbonylation to give derivatives with a phosphaalkene ligand in a novel μ-κ^2^:η^2^ coordination mode. However, reactions with organic azides involved [2 + 1] cycloaddition of the terminal nitrogen to the Mo−P double bond of the parent complex and coordination of the distal NR nitrogen without decarbonylation to give phosphatriazadiene derivatives, which evolved differently depending on R and M, to give either phosphaimine or triazenylphosphanyl derivatives.

## Introduction

**Chart 1:**
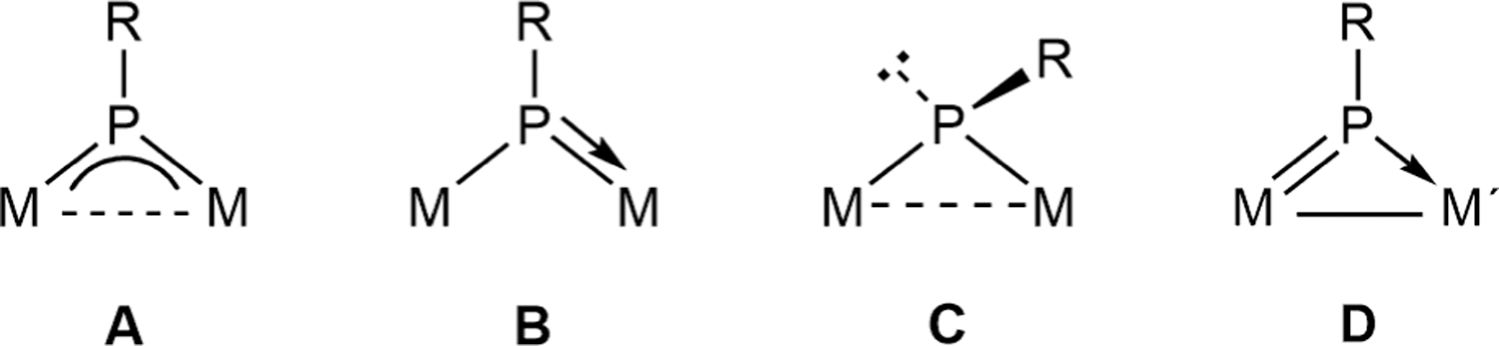
Coordination Modes of PR Ligands at Binuclear Complexes

Reactions of mononuclear metal complexes bearing terminal phosphinidene ligands (PR) with small organic molecules have proved to be a very successful and largely exploitable strategy to build a great variety of organophosphorus molecules on the coordination sphere of metal atoms.^1,2^ In contrast, only more recently this sort of reactivity has been extended to binuclear complexes featuring bridging PR ligands, to show that the particular coordination mode of the ligand (**A** to **C** in Chart 1) greatly influences the result of these reactions.^3^ The latter work, however, focused mostly on homometallic complexes, but the synergic and cooperative effects that the combination of distinct metals with different coordination surroundings can induce, as found in heterometallic complexes,^4^ have not been explored for phosphinidene-bridged complexes. Following our recent preparation of the novel heterometallic complex [MoReCp(μ-PR*)(CO)_6_] (**1a**),^5^ we found that, in spite of the isoelectronic nature of the Mo and Re fragments, the π-bonding metal–phosphorus interaction in this molecule is essentially located at the Mo–P junction, whereas bonding of P with the group 7 metal atom can be essentially described as a donor single bond, all of it accounting for a new coordination mode of the bridging phosphinidene ligand (**D** in Chart 1) deserving some studies about the chemical behavior derived from it. Preliminary studies on the reactivity of **1a** revealed, among other features, a defined trend of this complex to undergo cycloaddition processes at the Mo–P double bond when reacting with organic molecules having C–C or C–N triple bonds, such as alkynes and isocyanides.^5^ In this paper, we analyze the reactivity of **1a** and that of its manganese analogue [MoMnCp(μ-PR*)(CO)_6_] (**1b**)^6^ toward some small molecules having N–N multiple bonds, such as diazoalkanes and organic azides.

**Scheme 1:**
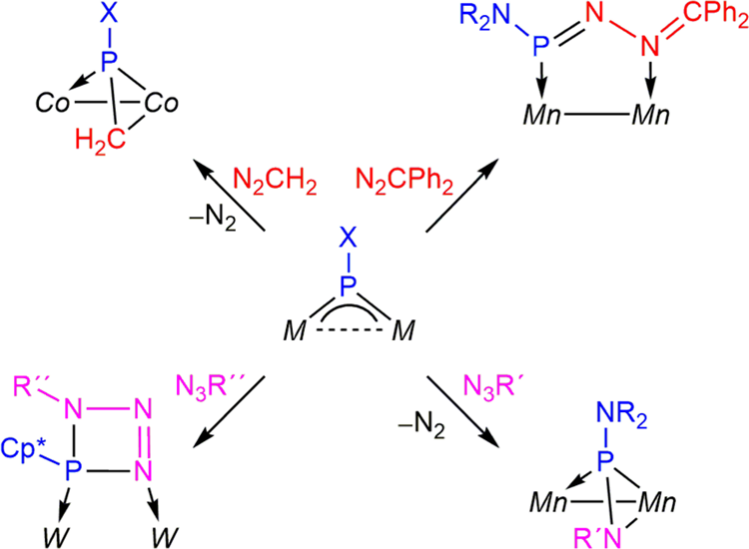
Diazoalkane and Azide Derivatives of Phosphinidene Complexes of type A *Mn*–Mn = Mn_2_(CO)_8_; *Co*–Co = Co_2_(CO)_4_(μ-Ph_2_PCH_2_PPh_2_); *W* = W(CO)_5_; X = Cp* or NR_2_, with Cp* = C_5_Me_5_ and R = ^*i*^Pr; R′= SiMe_3_, SnMe_3_, Ph, adamantyl; R″ = Cy, Hex.

Previous studies on reactions of homometallic PR-bridged complexes with diazoalkanes and organic azides are scarce, yet they indicate that both the coordination mode of the PR ligand and the particular metals involved in each case would have a significant influence on the output of these reactions, not only on the coordination mode of the newly generated organophosphorus ligands, but also on the denitrogenation processes that might follow at the complexes first formed in these reactions. The type **A** trigonal phosphinidene complexes [Mn_2_(μ-PN^*i*^Pr_2_)(CO)_8_] and [Co_2_(μ-PN^*i*^Pr_2_)(CO)_4_(μ-Ph_2_PCH_2_PPh_2_)] yielded phosphadiazadiene-, phosphaalkene- or phosphaimine-bridged derivatives upon reaction with diazoalkanes and azides, depending on the particular metal and reagent^7^ (Scheme 1). Phosphadiazadiene and phosphaalkene-bridged complexes were also formed in the reactions of the type **A** ditungsten complex [W_2_(μ-PCp*)(CO)_10_] with diazoalkanes,^8^ but no denitrogenation took place in reactions with azides, these yielding triazaphosphete-bridged complexes instead.^9^

**Scheme 2:**
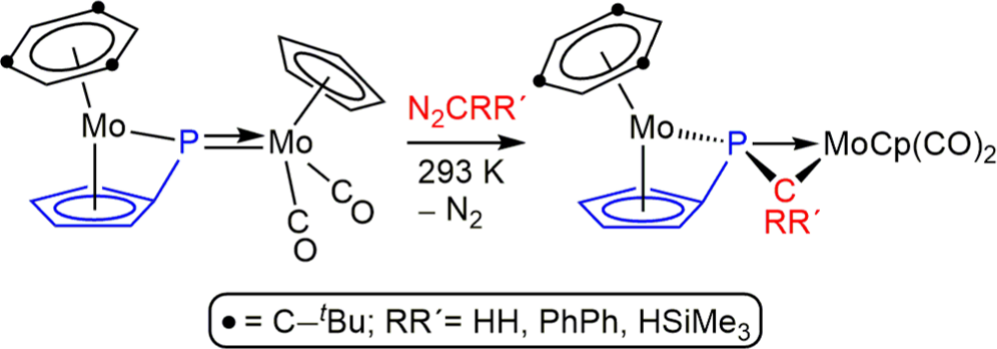
Diazoalkane Derivatives of a Dimolybdenum Complex of Type B

As for complexes of type **B**, only reactions of the dimolybdenum complex [Mo_2_Cp(μ-κ^1^:κ^1^,η^5^-PC_5_H_4_)(CO)_2_(η^6^-R*H)] with diazoalkanes have been investigated, these invariably resulting in spontaneous denitrogenation at room temperature to yield phosphaalkene-bridged products, which can be viewed as resulting from a formal [2 + 1] cycloaddition of the corresponding carbene to the Mo–P double bond of the parent complex (Scheme 2).^10^

**Scheme 3:**
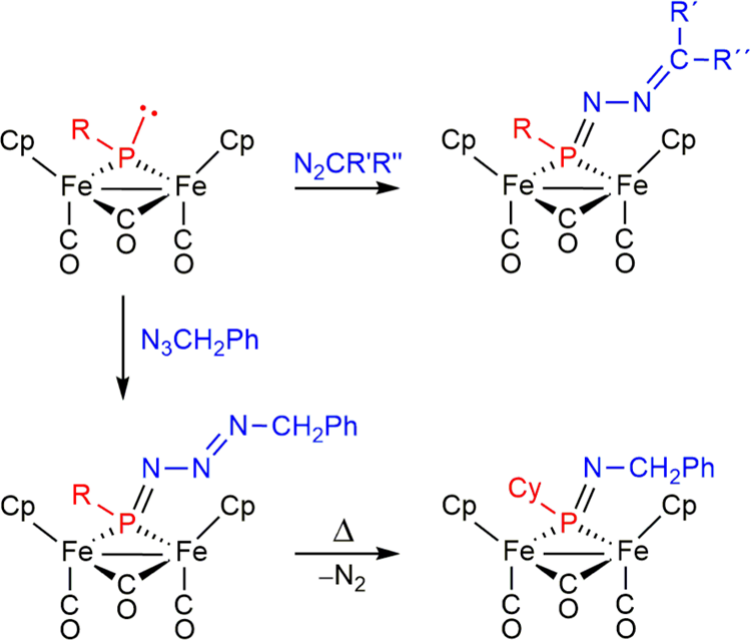
Diazoalkane and Azide Derivatives of Diiron Complexes of Type C R = Cy, Ph, R*; R′R″ = HH, HSiMe_3_, HCO_2_Et, PhPh.

In contrast, denitrogenation seems to be much less favored in reactions with complexes bearing pyramidal phosphinidene ligands (type **C**). Thus, the diiron complexes [Fe_2_Cp_2_(μ-PR)(μ-CO)(CO)_2_] (R = Cy, Ph, R*) reacted with different diazoalkanes and benzyl azide to give isolable κ^1^_P_:κ^1^_P_-phosphadiazadiene- and phosphatriazadiene-bridged derivatives, respectively, with denitrogenation being only induced in the latter case upon heating (Scheme 3).^11−13^ In the same line, we reported recently that the dimolybdenum complex [Mo_2_Cp(μ-κ^1^:κ^1^,η^5^-PC_5_H_4_)(CO)_2_(η^6^-R*H)(PMe_3_)] reacts with different diazoalkanes and organic azides at low temperature to give the corresponding κ^1^_P_:κ^1^_P_-phosphadiazadiene- and phosphatriazadiene-bridged derivatives, which, however, were quite unstable and could be only isolated as solid materials after protonation or methylation steps. Once again, denitrogenation was only observed in some of the azide derivatives.^14^ As we will discuss below, the reactions of the heterometallic complexes **1a,b** with diazoalkanes and organic azides reported here not only bear some similarities with the ones discussed above but also reflect a relevant role of the heterometallic center, whereby rare or new coordination modes of phosphaalkene, phosphatriazadiene, phosphaimine, and triazenylphosphanyl ligands are unveiled.

## Results and Discussion

### Reactions with Diazoalkanes

**Scheme 4:**
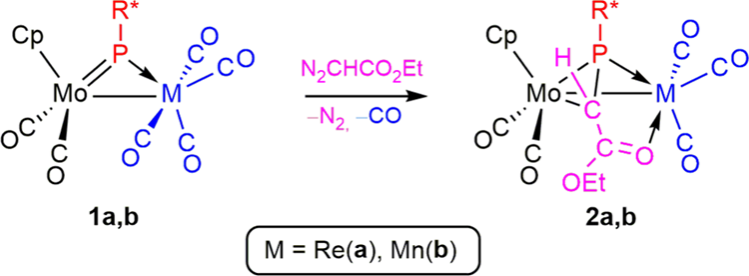
Preparation of Compounds **2**

The rhenium complex **1a** reacted slowly with a slight excess of ethyl diazoacetate at room temperature to give the phosphaalkene-bridged complex [MoReCp(μ-η^2^_P,C_:κ_^2^P,O_-PR*CHCO_2_Et)(CO)_5_] (**2a**) (Scheme 4) in modest yield (ca. 20% after chromatographic workup). Unfortunately, the use of less activated diazoalkanes led to no new complexes; thus, the reaction of **1a** with N_2_CHSiMe_3_ at room temperature just resulted in decomposition of the diazoalkane, whereas reaction with N_2_CPh_2_ only proceeded in refluxing toluene, and then led to decomposition of both the metal complex and the added diazoalkane. The manganese complex **1b** seemed less reactive than its rhenium analogue, since its reaction with ethyl diazoacetate only proceeded at a significant rate upon warming the solution up to ca. 333 K, then needing several additions of the reagent to counterbalance the decomposition of the latter. Eventually, however, the corresponding phosphaalkene-bridged complex [MoMnCp(μ-η^2^_P,C_:κ^2^_P,O_-PR*CHCO_2_Et)(CO)_5_] (**2b**) could be isolated in medium yield (ca. 50% after chromatographic workup).

The formation of complexes **2** formally follows from a [2 + 1] cycloaddition of the carbene CHCO_2_Et to the Mo–P double bond of the parent complexes **1**, as observed in the reactions of the type **B** phosphinidene complex mentioned in the Introduction (Scheme 2), this now being followed by coordination of the carbonyl group of the reagent to block the vacant coordination position left by the spontaneous decarbonylation of the M(CO)_4_ fragment. The actual mechanism of this process, however, might actually be more complex and likely would involve first the formation of a P–N bond to give undetected phosphadiazadiene intermediates (cf. Scheme 3) that would rapidly evolve through denitrogenation, as actually observed in the reactions of **1a** with azides to be discussed later on, and then through a decarbonylation step that would force the formation of a new O–Re/Mn bond.

**Figure 1:**
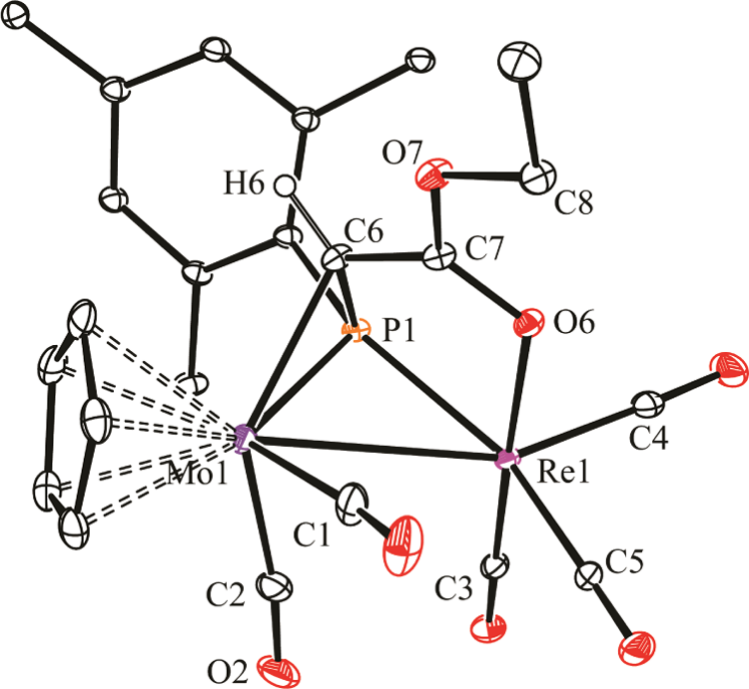
ORTEP diagram (30% probability) of compound **2a**, with ^*t*^Bu (except their C^1^ atoms) and most H atoms omitted for clarity. Only one of the two independent molecules in the unit cell is shown.

The structure of the rhenium complex **2a** in the crystal (Figure 1 and Table 1) can be derived from that of the parent compound **1a** by adding the carbenic carbon to the Mo–P double bond of the precursor through a direction perpendicular to the MoPRe plane, thus maximizing interaction with the π and π* frontier orbitals of **1a**,^5^ while the C=O oxygen atom in the carbene substituent binds the Re atom *trans* to a carbonyl ligand, thus defining a little twisted RePCCO five-membered ring. The resulting phosphaalkene ligand can thus be considered as contributing with two electrons to the Mo center (η^2^ coordination) and with four electrons to the rhenium center (κ^2^_P,O_ coordination). This yields a 34-electron complex for which a single Mo–Re bond has to be proposed according to the 18 electron rule, which is in agreement with the Mo–Re distance of 3.1579(3) Å, very similar to the distance of 3.1745(6) Å measured in the parent compound **1a**.^5^ We note that no other heterometallic complex with κ^1^:η^2^-bridging phosphaalkene ligands appears to have been crystallographically characterized so far,^15^ and the additional coordination of the oxygen atom to render a 6-electron donor μ-κ^2^:η^2^ coordination mode is also unprecedented for a phosphaalkene ligand.

**Table 1:** Selected Bond Lengths (Å) and Angles (°) for Compound **2a**

Mo1–Re1	3.1579(3)	Mo1–P1–Re1	81.17(2)
Mo1–P1	2.4520(7)	P1–Mo1–C1	109.9(1)
Mo1–C6	2.296(3)	P1–Mo1–C2	90.6(1)
Mo1–C1	1.962(3)	P1–Re1–C3	99.2(1)
Mo1–C2	1.999(3)	P1–Re1–C4	108.5(1)
Re1–P1	2.4016(7)	P1–Re1–C5	156.5(1)
Re1–C3	1.906(3)	P1–Re1–O6	81.5(1)
Re1–C4	1.905(3)	C1–Mo1–C2	86.9(2)
Re1–C5	1.932(3)	Mo1–C6–P1	72.7(1)
Re1–O6	2.203(2)	P1–C6–C7	117.0(2)
P1–C6	1.780(3)	Re1–O6–C7	115.7(2)
C7–O6	1.250(3)		

Spectroscopic data for compounds **2a** and **2b** in solution are consistent with the solid-state structure of **2a**. The IR spectra display, in each case, a high-frequency C–O stretch at ca. 2020 cm^–1^ of high intensity, as expected from the presence of pyramidal M(CO)_3_ oscillators,^16^ while evidence for the coordination of the carboxylate group is given by the significant red shift of the corresponding C=O stretch, from ca. 1700 cm^–1^ observed in uncoordinated groups to ca. 1550 cm^–1^ in complexes **2**. This is also consistent with the C–ORe distance of 1.250(3) Å measured for **2a** in the solid state, slightly enlarged when compared to the reference value of ca. 1.21 Å for a double bond between these atoms.^17^ The formation of the three-membered phosphametallacycle found in compounds **2** is denoted by the dramatic shielding of its ^31^P resonance, which moves from ca. 600 ppm (for the parent compounds **1**) to ca. 100 ppm (Table 2). As expected, the resonance of the Re compound appears some 50 ppm more shielded than the one of its Mn analogue, a difference typically observed when comparing chemical shifts of P atoms bound to heavier metals within the same group.^18^ Finally, we note that the methylenic group of the MoPC ring gives rise to considerably shielded ^13^C (δ_C_ ca. 25 ppm) and ^1^H (δ_H_ ca. 2 ppm) NMR resonances, which is consistent with the strong η^2^ coordination observed in the solid state, as deduced from the values of the Mo–C and C–P distances (2.296(3) and 1.780(3) Å respectively), which are close to the expected figures for single bonds between the C(sp^2^) and Mo or P atoms (2.27 and 1.80 Å).^19^

**Table 2:** Selected IR and ^31^P{^1^H} NMR Data for New Compounds^a^

compound	ν(CO)	δ (P)
[MoReCp(μ-η^2^_P,C_:κ^2^_P,O_-PR*CHCO_2_Et)(CO)_5_] (**2a**)	2022 (vs), 1949 (m), 1925 (m), 1905 (m), 1550 (w)	83.6
[MoMnCp(μ-η_^2^P,C_:κ^2^_P,O_-PR*CHCO_2_Et)(CO)_5_] (**2b**)	2018 (vs), 1946 (s), 1911 (m), 1869 (w), 1564 (w)	127.0
[MoReCp(μ-η^2^_P,N_:κ_^2^P,N′_-PR*N_3_Bn)(CO)_6_] (**3a.1**)	2102 (m), 2011 (vs), 1996 (m), 1959 (m), 1932 (m), 1848 (m)	–18.7
[MoReCp{μ-η^2^_P,N_:κ^2^_P,N′_-PR*N_3_(*p*-tol)}(CO)_6_] (**3a.2**)	2101 (m), 2012 (vs), 1997 (m), 1964 (m), 1933 (m), 1849 (m)	–22.6
[MoMnCp(μ-η^2^_P,N_:κ^2^_P,N′_-PR*N_3_Bn)(CO)_6_] (**3b.1**)	2086 (m), 2014 (vs), 2000 (s), 1964 (m), 1932 (m), 1849 (m)	31.8
[MoReCp{μ-η^2^_P,N_:κ^2^_P,N′_-PR*N_3_(*p*-tol)}(CO)_6_] (**3b.2**)	2086 (s), 2012 (vs), 2002 (vs), 1971 (m), 1932 (m), 1849 (m)	25.1
[MoReCp{μ-κ_P_:κ_N_-PR*N(*p*-tol)}(CO)_6_] (**4a**)	2072 (m), 1979 (vs), 1964 (m), 1933 (s), 1922 (sh, m), 1886 (m)	289.6
[MoMnCp{μ-κ_P_:κ_N_-PR*N(*p*-tol)}(CO)_6_] (**4b**)	2049 (m), 1971 (vs), 1960 (sh, w), 1935 (s), 1922 (m, sh), 1887 (w)	292.6
[Mn(κ_^2^P,N_-PR*NHNNBn)(CO)_3_] (**5.1**)	2000 (vs), 1912 (s)	252.6
[Mn{κ_^2^P,N_-PR*NHNN(*p*-tol)}(CO)_3_] (**5.2**)	2002 (vs), 1914 (s)	253.9

a IR spectra recorded in dichloromethane solution; NMR spectra recorded in CD_2_Cl_2_ solution at 121.48 MHz and 293 K, with chemical shifts (δ) in ppm relative to external 85% aqueous H_3_PO_4_.

### Reactions of the Rhenium Complex **1a** with Organic Azides

**Scheme 5:**
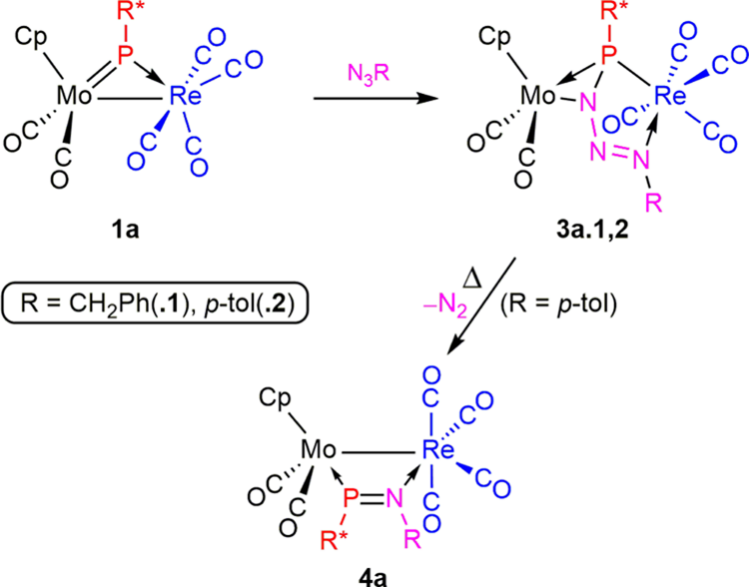
Reactions of 1a with Organic Azides

Compound **1a** reacted with benzyl- or *p*-tolyl azide smoothly at room temperature in toluene solution to give the green complexes [MoReCp(μ-η^2^_P,N_:κ_^2^P,N′_^2^-PR*N_3_R)(CO)_6_] [R = Bn (**3a.1**), *p*-tol (**3a.2**)] in good yields (ca. 80% after chromatographic workup), which display bridging phosphatriazadiene ligands in a novel 6-electron donor μ-κ^2^:η^2^ coordination mode (Scheme 5).^20^ Compounds **3a** formally follow from a [2 + 1] cycloaddition of the terminal N atom of the azide to the Mo–P double bond of the parent complex, this being followed by the coordination of the distal NR nitrogen to the rhenium center without decarbonylation. As a result of the latter coordination, the intermetallic interaction is vanished in the resulting 36 electron complexes. The contrast with the diazoalkane reactions of **1a,b** discussed above is thus evident, since no spontaneous denitrogenation takes place here at room temperature. In fact, the benzyl azide derivative **3a.1** could be heated in refluxing toluene solution without significant transformation. However, the *p*-tolyl azide derivative **3a.2** underwent clean denitrogenation upon heating in toluene solution at 333 K to yield the orange complex [MoReCp{μ-κ_P_:κ_N_-PR*N(*p*-tol)}(CO)_6_] (**4a**) in good yield (ca. 70% yield after chromatographic workup). The latter displays a bridging phosphaimine (or iminophosphinidene) ligand in a rare κ_P_:κ_N_ coordination mode (Scheme 5), previously identified only in the rhenium complex [Re_2_(μ-Br)_2_{μ-κ_P_:κ_N_-P(N^*i*^Pr_2_)N(^*t*^Bu)}(CO)_6_].^21^ We note the specific ligation of P to the Mo atom and the NR nitrogen to the Re atom, thus retaining most of the connections in the parent compound **3a.2**. No evidence for the formation of an alternative isomer of **4a** (with *P*-binding of the bridging ligand to Re, and *N*-binding to Mo) was obtained in this denitrogenation reaction.

From the above differences between the thermal evolution of compounds **3a.1** and **3a.2**, we may conclude that their phosphatriazadiene ligands are more prone to denitrogenation when having an electron-withdrawing aryl substituent at the N atom rather than an electron-releasing alkyl one. This is in line with our previous studies on the reactions of the dimolybdenum complex of type **C** [Mo_2_Cp(μ-κ^1^:κ^1^,η^5^-PC_5_H_4_)(CO)_2_(η^6^-R*H)(PMe_3_)] with different organic azides.^14^ Thus, it seems that denitrogenation processes in bridging phosphatriazadiene ligands are largely influenced by the nature of the substituent at the N atom (electron-withdrawing or electron-releasing ones) and less by the exact nature of the dimetal site to which they are bound.

### Structure of Phosphatriazadiene Complexes **3a**

**Figure 2:**
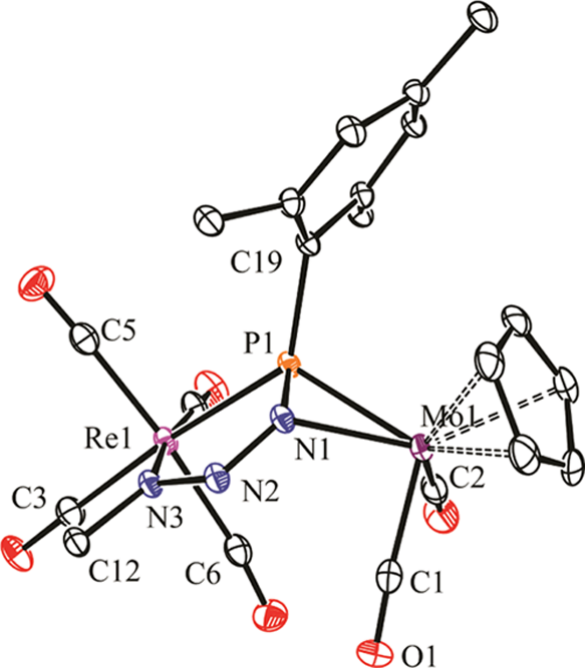
ORTEP diagram (30% probability) of compound **3a.2**, with ^*t*^Bu (except their C^1^ atoms) and H atoms omitted for clarity.

The molecule of the *p*-tolyl azide derivative **3a.2** in the crystal (Figure 2 and Table 3) can be derived from that of the parent compound upon [2 + 1] cycloaddition of the terminal N atom of the azide to the Mo–P double bond of the parent compound, expectedly from a direction perpendicular to the former MoPRe plane, as discussed above. The coordination of the distal NR atom to the Re center removes the intermetallic interaction (Mo1···Re1 ca. 4.36 Å) and increases the coordination number of this metal atom, which now displays a local octahedral geometry, whereas the conformation of the resulting PNNNRe five-membered ring is essentially planar. The M–P and M–N lengths are not unusual for the single bonds to be formulated for these connections, so it is the P1–N1 length of 1.734(3) Å, only a bit below the reference figure of 1.78 Å for a single bond between these atoms.^19^ Unexpectedly, however, the N–N lengths are similar to each other, with a value of ca. 1.30 Å, which is intermediate between the reference figures for N–N and N=N bonds (1.42 and 1.25 Å, respectively),^17,19^ even if the pyramidal environment around the bridgehead N1 atom would seem to disfavor any delocalization of the N2–N3 π interaction. We should finally remark that compound **3a.2** provides the first structural characterization of the μ-η^2^_P,N_:κ_P_ (and μ-η^2^_P,N_:κ_^2^P,N′_) coordination mode for a bridging phosphatriazadiene ligand. As concluded from the above geometrical analysis, the η^2^ interaction of the P=N bond of this ligand with the Mo atom is strong enough to get close to the “metallacyclopropane” extreme of [2 + 1] cycloadditions.

**Table 3:** Selected Bond Lengths (Å) and Angles (°) for Compound **3a.2**

Mo1···Re1	4.3629(5)	Mo1–P1–Re1	122.28(3)
Mo1–P1	2.4964(8)	Mo1–P1–N1	58.51(9)
Mo1–N1	2.172(3)	Mo1–N1–P1	78.6(1)
Mo1–C1	1.949(4)	P1–N1–N2	122.7(2)
Mo1–C2	1.959(4)	N1–N2–N3	117.7(3)
Re1–P1	2.4850(7)	N2–N3–Re1	122.6(2)
Re1–N3	2.201(3)	P1–Re1–N3	75.52(7)
P1–N1	1.734(3)	P1–Re1–C3	169.8(1)
N1–N2	1.313(4)	P1–Re1–C5	94.5(1)
N2–N3	1.301(4)	C1–Mo1–C2	77.2(2)

Spectroscopic data for compounds **3a.1** and **3a.2** in solution are consistent with the solid-state structure of **3a.2**. In particular, the IR spectra of these compounds display, in each case, six C–O stretches with a pattern indicative of the presence of vibrationally independent cisoid Mo(CO)_2_ and disphenoidal M(CO)_4_ oscillators, as expected for molecules having these fragments not connected through a metal–metal bond.^16^ In addition, the ^31^P NMR resonances of these complexes appear strongly shielded (δ_P_ ca. −20 ppm), with a chemical shift some 100 ppm below the one measured for the phosphaalkene-bridged complex **2a**, which likely is another spectroscopic feature derived from the absence of a metal–metal bond connecting the metal fragments in these molecules.^18^ Other spectroscopic parameters of these complexes are not unusual and deserve no detailed comments.

### Structure of the Phosphaimine Complex **4a**

**Figure 3:**
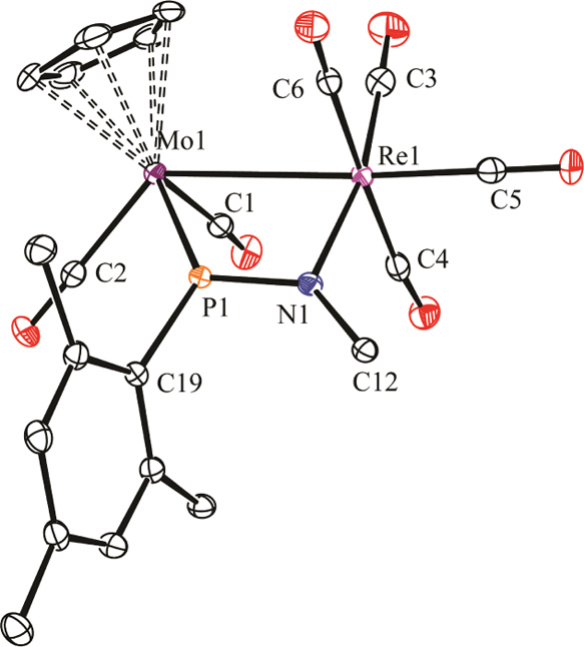
ORTEP diagram (30% probability) of compound **4a**, with ^*t*^Bu and *p*-tol groups (except their C^1^ atoms) and H atoms omitted for clarity.

The molecule of this complex in the crystal (Figure 3 and Table 4) is built from cisoid MoCp(CO)_2_ and disphenoidal Re(CO)_4_ fragments connected via a 4-electron donor phosphaimine ligand, which is *P*-bound to the Mo atom and *N*-bound to the Re atom, and defines an almost flat MoReNP central ring. The coordination sphere of the metal atoms is completed with an intermetallic single bond, as expected for a 34-electron complex. The corresponding length of 3.0968(2) Å is a bit shorter than the ones in compounds **1a** or **2a** but otherwise is not unusual for a single bond between these metal atoms (cf. 3.1307(8) Å in [MoRe(μ-η^5^:κ^1^_P_-C_5_H_4_PCy_2_)(CO)_7_]).^22^

**Table 4:** Selected Bond Lengths (Å) and Angles (°) for Compound **4a**

Mo1–Re1	3.0968(2)	Mo1–Re1–N1	77.89(6)
Mo1–P1	2.3081(7)	Re1–Mo1–P1	63.40(2)
Mo1–C1	1.968(3)	Mo1–P1–N1	119.9(1)
Mo1–C2	1.961(3)	P1–N1–Re1	98.8(1)
Re1–N1	2.205(2)	P1–Mo1–C1	98.4(1)
Re1–C3	1.936(3)	P1–Mo1–C2	78.6(1)
Re1–C4	1.995(3)	N1–Re1–C3	168.7(1)
Re1–C5	1.918(3)	N1–Re1–C4	93.4(1)
P1–N1	1.603(2)	C1–Mo1–C2	83.0(1)

**Chart 2:**
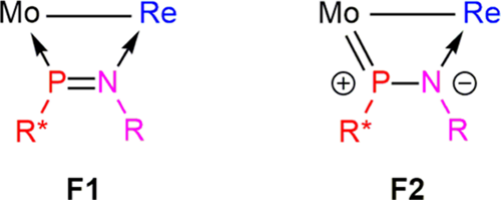
Canonical Forms Proposed for Compound **4a**

As for the coordination of the bridging ligand, we note that the Re–N separation of 2.205(2) Å is close to the reference figure of ca. 2.22 Å for a single bond between these atoms,^19^ actually very close to the corresponding distance measured in the mentioned complex [Re_2_(μ-Br)_2_{μ-κ_P_:κ_N_-P(N^*i*^Pr_2_)N(^*t*^Bu)}(CO)_6_] (2.21(2) Å).^21^ In contrast, the Mo–P distance of 2.3081(7) Å is significantly shorter than the values of around 2.45 Å typically found for phosphines bound to molybdenum (for instance, ca. 2.44 Å in [Mo_2_Cp_2_(CO)_4_(μ-Ph_2_PCH_2_PPh_2_)]),^23^ it being actually comparable to the values of ca. 2.31 Å measured in the type **A** phosphinidene complex [Mo_2_Cp_2_(μ-PR*)(CO)_4_],^24^ a molecule for which a Mo–P bond order of 1.5 should be proposed. At the same time, the P–N distance of 1.603(2) Å is somewhat longer than the reference value of 1.57 Å for a double bond between these atoms or the distance of 1.56(2) Å measured in the mentioned dirhenium complex. All of this indicates that for our heterometallic complex **4a.2** (but not for the mentioned Re_2_ complex), the interaction of the phosphaimine ligand with the heterometallic center cannot be just described using conventional single-donor bonds (canonical form **F1** in Chart 2). Instead, the above geometrical parameters suggest that there must also be a significant contribution of the interaction represented by canonical form **F2**, involving a Mo–P double bond and a P–N single bond.

Spectroscopic data for **4a** in solution imply a symmetry higher than the one found in the crystal, thus suggesting the presence of dynamic effects. Its IR spectrum displays six C–O stretches, and the medium intensity of the most energetic band at 2072 cm^–1^ is characteristic of M(CO)_4_ fragments with local disphenoidal geometry.^16^ On the other hand, its ^31^P NMR spectrum displays a substantially deshielded resonance, with a chemical shift (δ_P_ 289.6 ppm) comparable to the one measured for the mononuclear phosphanyl complex *syn*-[MoCp(PR*Cl)(CO)_2_] (*δ*_P_ 266.8 ppm),^5^ in any case consistent with the trigonal environment of the P atom of the ligand. However, the ^13^C and ^1^H NMR spectra of **4a** display single resonances for each of the pairs of Mo-bound carbonyls, two Re-bounds carbonyls, ring protons, and *ortho*-^*t*^Bu protons of the supermesityl group (see the Experimental Section). This suggests the operation of a dynamic process involving fast rearrangement (on the NMR time scale) of the MoCp(CO)_2_ fragment, so that the Cp (and CO ligands) exchange positions on both sides of the MoReNP plane, a dynamic process well-known for the MoCp(CO)_2_ fragments in tetracarbonyl complexes of type [Mo_2_Cp_2_(μ-H)(μ-PRR′)(CO)_4_],^25^ and not investigated here.

### Reactions of the Manganese Complex **1b** with Organic Azides

**Scheme 6:**
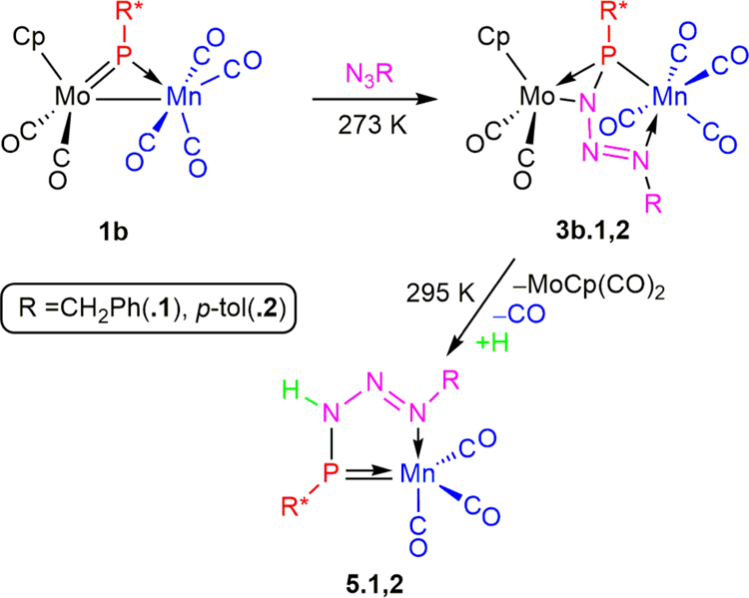
Reactions of **1b** with Organic Azides

Compound **1b** turned out to be more reactive toward organic azides than its Re analogue **1a**, as it was able to react with benzyl or *p*-tolyl azide, even at 273 K, to give the corresponding phosphatriazadiene-bridged derivatives [MoMnCp(μ-η^2^_P,N_:κ^2^_P,N′_-PR*N_3_R)(CO)_6_] [R = Bn (**3b.1**), *p*-tol (**3b.2**)] as major products, respectively (Scheme 6). The latter complexes could be isolated as green solids in ca. 55% yield upon chromatographic purification as long as all manipulations were carried out at low temperatures, as they were thermally unstable, and their spectroscopic data (Table 2 and Experimental Section) were analogous to those of the rhenium complexes **3a.1,2**, thus indicating that they all display the same structure.

Upon standing in solution at room temperature, the benzyl azide derivative **3b.1** fully degraded in a few hours to give the mononuclear phosphanyl complex [Mn(κ^2^_P,N_-PR*NHNNBn)(CO)_3_] (**5.1**) as a major product, which could be isolated in 58% yield upon chromatographic workup. The *p*-tolyl azide derivative **3b.2** behaved similarly and yielded the related complex [Mn{κ_^2^P,N_-PR*NHNN(*p*-tol)}(CO)_3_] (**5.2**) as a major product (44% yield after purification), but in this case, small amounts of the phosphaimine-bridged complex [MoMnCp{μ-κ_P_:κ_N_-PR*N(*p*-tol)}(CO)_6_] (**4b**) were also formed. Compound **4b** obviously follows from a minor denitrogenation pathway of **3b.2** that was dominant for its rhenium analogue **3a.2** and is also taking place for the azide derivative having the electron-withdrawing aryl substituent. Spectroscopic data for this product are analogous to those of the rhenium complex **4a** and deserve no particular comment, except for the observation that its ^31^P chemical shift of 292.6 ppm is almost identical to the one measured for **4a** (δ_P_ 289.6 ppm). This indicates that the coordination mode of the phosphaimine ligand also involves, in this case, the selective coordination of phosphorus to the Mo atom.

The formation of compounds **5** requires the cleavage of Mo–P and Mo–N bonds in the parent complexes **3b**. The latter cleavage would generate MoCp(CO)_2_ and Mn(CO)_4_(RPPN_3_R) radicals that would evolve differently. The Mo fragment appears to just decompose, as no likely derivatives of it ([Mo_2_Cp_2_(CO)*_n_*], with *n* = 4, 6, or [MoCpH(CO)_3_]) were identified in the corresponding reaction mixtures. As for the Mn fragment, it would undergo decarbonylation and H atom abstraction (from the solvent or trace amounts of water present in the solvent), both of them being well-established reactions of organometallic radicals.^26^ The abstraction process would occur selectively at the P-bound nitrogen of the phosphatriazadiene group to eventually render a triazenylphosphanyl ligand, although the exact sequence of events (degradation/decarbonylation/abstraction) is unknown. Under this view, the fact that the related rhenium complexes **3a** are not degrading thermally into similar mononuclear complexes can possibly be attributed to the higher reluctance of the Re(CO)_4_ fragment (compared to the Mn(CO)_4_ one) to undergo decarbonylation, likely at some of the early stages preceding degradation into mononuclear fragments.

### Structure of Triazenylphosphanyl Complexes **5**

**Figure 4:**
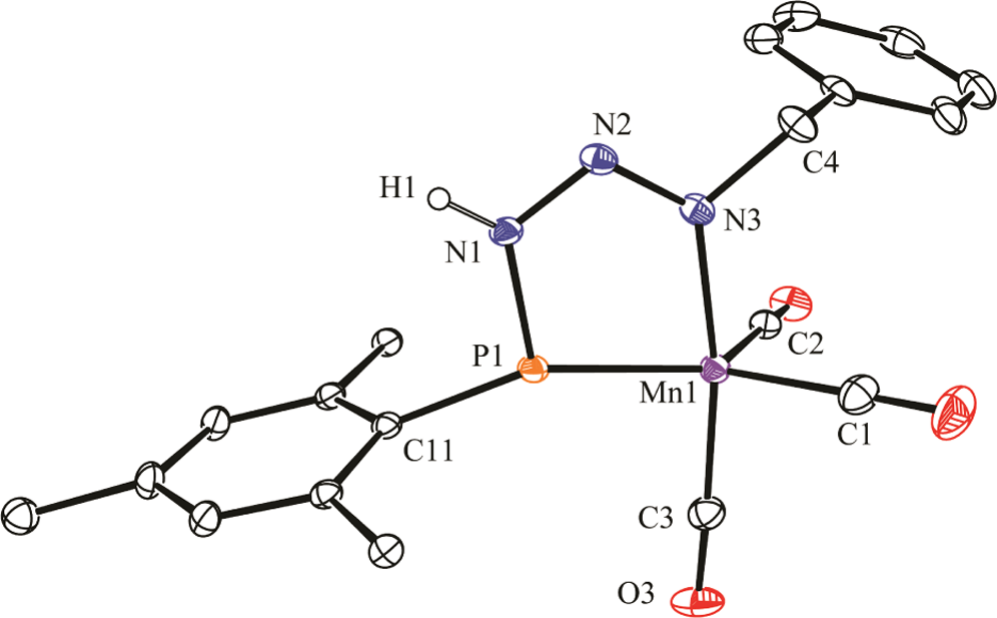
ORTEP diagram (30% probability) of compound **5.1**, with ^*t*^Bu (except its C^1^ atoms) and most H atoms omitted for clarity.

In the crystal (Figure 4 and Table 5), the benzyl azide derivative **5.1** displays a distorted trigonal bipyramidal environment for the manganese atom, with three carbonyl ligands occupying two equatorial and one axial position, while the bidentate triazenylphosphanyl ligand binds the metal atom through its phosphanyl group at the remaining equatorial site, and through the distal NBn nitrogen at the remaining axial site, *trans* to a carbonyl ligand (C3–Mn1–N3 ca. 170°). The ligand thus configures an almost perfectly flat five-membered MnPN_3_ ring that bisects the Mn(CO)_3_ fragment and defines a trigonal planar environment around the P atom but with angles strongly departing from the ideal values of 120° (cf. Mn1–P–C11 = 152.8(1)°). We note that no other complex with a chelating, 5-electron donor triazenylphospanyl ligand appears to have been structurally characterized so far, although we have reported previously examples of a similar ligand *bridging* molybdenum atoms in a μ-κ_^1^P_:κ^2^_P,N_ fashion.^14^ The corresponding Mn–N length of 1.994(2) Å in **5.1** falls on the short side of the range of distances measured in imine and related carbonyl complexes of manganese (1.95–2.15 Å),^27^ while the short Mn–P distance of 2.1168(6) Å is consistent with the formulation of a double bond for the interaction of this three-electron donor group with the manganese atom, this being slightly shorter than the value of 2.1414(8) Å measured in the MoMn complex [MoMnCp(μ-κ^1^:κ^1^,η^6^-PR*)(CO)_4_],^28^ and falling in the upper part of the range of ca. 2.06–2.12 Å found for the few mononuclear complexes of type [Mn(PX_2_)(CO)_4_] structurally characterized to date (X = R, OR, NR_2_ substituent).^29,30^ As for other distances within the phosphametallacyclic ring, we note that the P1–N1 separation of 1.704(2) Å is below the reference value for a P–N single bond (ca. 1.78 Å), while the N1–N2 and N2–N3 separations (1.325(2) and 1.297(2) Å, respectively) have values close to each other and intermediate between the reference values for single and double bonds between N atoms (1.42 and 1.25 Å respectively),^17,19^ all of this suggesting significant delocalization of π-bonding interactions along the PN_3_ chain.

**Table 5:** Selected Bond Lengths (Å) and Angles (°) for Compound **5.1**

Mn1–P1	2.1168(6)	P1–Mn1–C1	141.7(1)
Mn1–N3	1.994(2)	P1–Mn1–C2	121.2(1)
Mn1–C1	1.811(2)	P1–Mn1–C3	91.1(1)
Mn1–C2	1.803(2)	P1–Mn1–N3	79.17(5)
Mn1–C3	1.800(2)	C3–Mn1–N3	169.8(1)
P1–N1	1.704(2)	Mn1–P1–N1	103.9(1)
N1–N2	1.325(2)	Mn1–P1–C11	152.8(1)
N2–N3	1.297(2)	P1–N1–N2	117.8(1)
		N1–N2–N3	111.9(2)
		N2–N3–Mn1	127.2(1)
		C1–Mn1–C2	97.1(1)

Spectroscopic data for compounds **5.1** and **5.2** in solution are consistent with the solid-state structure of **5.1**. Thus, their IR spectra display, in each case, two strong C–O stretches characteristic of pyramidal M(CO)_3_ oscillators in a high-symmetry local enviroment,^16^ while the presence of a N-bound hydrogen is revealed by a weak N–H stretch at 3329 cm^–1^ in the solid-state spectrum of **5.1** and by ^1^H NMR resonances at ca. 11 ppm in both cases. Both compounds display quite deshielded ^31^P NMR resonances at ca. 253 ppm, indicative of the retention of the trigonal environment for the P atom observed in the solid state (cf. 246 ppm for [Mn{P(RNC_2_H_2_NR)}(CO)_4_], with R = 2,6-C_6_H_3_^*i*^Pr_2_).^30c^ We finally note that rather than the separate resonances expected for the equatorial and axial carbonyls of these molecules, the ^13^C NMR spectra of compounds **5.1,2** displayed single-carbonyl resonances at ca 227 ppm in each case when recorded at room temperature or 253 K. This reveals the operation of a fast (on the NMR time scale) carbonyl exchange process within the Mn(CO)_3_ fragment, quite common in carbonyl complexes having pyramidal M(CO)_3_ fragments, which was not further investigated.

## Concluding Remarks

Reactions of the heterometallic phosphinidene complexes **1a,b** with ethyl diazoacetate invariably involved denitrogenation of the organic molecule and spontaneous decarbonylation of the group 7 metal fragment, thus forcing the resulting phosphaalkene ligand to adopt a novel 6-electron donor μ-κ^2^:η^2^ coordination mode following coordination of the O(carbonyl) atom of the organic substituent. In contrast, reactions with benzyl- or *p*-tolyl azide involved first [2 + 1] cycloaddition of the terminal N atom of the azide to the Mo–P double bond of the parent complex, this being followed by coordination of the distal NR nitrogen to the group 7 metal fragment without decarbonylation, to give complexes [MoMCp(μ-η^2^_P,N_:κ^2^_P,N′_-PR*N_3_R)(CO)_6_], which display bridging phosphatriazadiene ligands in a novel 6-electron donor μ-κ^2^:η^2^ coordination mode. These complexes, however, evolved differently, depending on the substituent R and M. The rhenium complexes did not degrade into mononuclear species, possibly because of their higher reluctance to undergo decarbonylation processes, and only when having the electron-withdrawing aryl substituent, they could be denitrogenated upon heating to give [MoReCp{μ-κ_P_:κ_N_-PR*N(*p*-tol)}(CO)_6_], which displays a phosphaimine ligand in a rare κ_P_:κ_N_ coordination mode, with substantial multiplicity in the corresponding Mo–P bond. The manganese complexes, however, degraded spontaneously at room temperature to give the corresponding mononuclear complex [Mn(κ^2^_P,N_-PR*NHNNR)(CO)_3_], which provides the first examples of a chelating, 5-electron donor triazenylphosphanyl ligand structurally characterized so far, with the P atom occupying an equatorial site and connected with the manganese atom through a Mn–P double bond.

## Experimental Section

### General Procedures and Starting Materials

General experimental procedures, as well as the preparation of compounds [MoReCp(μ-PR*)(CO)_6_] (**1a**) and [MoMnCp(μ-PR*)(CO)_6_] (**1b**), were carried out as described previously (Cp = η^5^-C_5_H_5_; R* = 2,4,6-C_6_H_2_^*t*^Bu_3_).^5,6^

#### Preparation of [MoReCp(μ-η^2^_P,C_:κ_^2^P,O_-PR*CHCO_2_Et)(CO)_5_] (**2a**)

Ethyl diazoacetate (5 μL, 0.048 mmol) was added to a toluene solution (6 mL) of compound **1a** (0.025 g, 0.032 mmol), and the mixture was stirred at room temperature for 18 h. A second addition of the reagent was then made (5 μL, 0.048 mmol), and stirring was continued for 1 h to give a brown-orange solution. The solvent was then removed under vacuum, the residue was extracted with dichloromethane/petroleum ether (1/10), and the extracts were chromatographed on alumina at 258 K. Elution with the same solvent mixture gave a yellow fraction, yielding, after removal of solvents, compound **2a** as a yellow microcrystalline solid (0.005 g, 19%). Crystals of **2a** for the X-ray study were grown from a diethyl ether/petroleum solution at 253 K. Anal. calcd for C_32_H_40_MoO_7_PRe: C, 45.23; H, 4.74. Found: C, 44.95; H, 4.30. ^1^H NMR (400.13 MHz, CD_2_Cl_2_): δ 7.31, 7.24 (2s, br, 2 × 1H, C_6_H_2_), 4.84 (s, 5H, Cp), 4.26, 4.18 (2m, 2 × 1H, OCH_2_), 2.41 (s, 1H, CH), 1.63, 1.46, 1.30 (3s, 3 × 9H, ^*t*^Bu), 1.25 (t, *J*_HH_ = 7.1, 3H, CH_3_). ^13^C{^1^H} NMR (100.63 MHz, CD_2_Cl_2_): δ 235.6 (s, MoCO), 228.1 (d, *J*_CP_ = 6, MoCO), 200.8 (s, ReCO), 199.5 (d, *J*_CP_ = 6, ReCO), 198.5 (s, *C*O_2_Et), 195.5 (d, *J*_CP_ = 5, ReCO), 160.1 [s, C^4^(C_6_H_2_)], 159.6 [d, *J*_CP_ = 7, C^2,6^(C_6_H_2_)], 150.9 [s, C^6,2^(C_6_H_2_)], 129.0 [s, br, C^1^(C_6_H_2_)], 124.2 [d, *J*_CP_ = 8, C^3,5^(C_6_H_2_)], 121.2 [d, *J*_CP_ = 11, C^5,3^(C_6_H_2_)], 91.3 (s, Cp), 64.2 (s, OCH_2_), 40.8, 39.7, 35.0 [3s, C^1^(^*t*^Bu)], 34.4 [s, C^2^(^*t*^Bu)], 33.5 [d, *J*_CP_ = 4, C^2^(^*t*^Bu)], 31.1 [s, C^2^(^*t*^Bu)], 26.0 (d, *J*_CP_ = 9, CH), 14.7 (s, CH_3_).

#### Preparation of [MoMnCp(μ-η^2^_P,C_:κ^2^_P,O_-PR*CHCO_2_Et)(CO)_5_] (**2b**)

Ethyl diazoacetate (4 μL, 0.039 mmol) was added to a toluene solution (8 mL) of compound **1b** (0.020 g, 0.030 mmol), and the mixture was stirred at 333 K for 4 h to give a brown solution. Ethyl diazoacetate (4 μL, 0.039 mmol) was again added to the latter solution, and stirring was continued for another 4 h. This operation was repeated one more time to eventually give an orange solution. Workup as for **2a** (elution with a 1/12 mixture) gave a minor fraction of unreacted **1b**. Elution with a 1/10 mixture gave a minor yellow fraction containing small amounts of [MoMnCp(μ-H){μ-P(CH_2_CMe_2_)C_6_H_2_^*t*^Bu_2_}(CO)_6_].^6^ Finally, elution with a 1/6 mixture gave a major yellow fraction, yielding compound **2b** as a yellow microcrystalline solid (0.012 g, 56%). Anal. calcd for C_32_H_40_MnMoO_7_P: C, 53.49; H, 5.61. Found: C, 53.25; H, 5.19. ^1^H NMR (400.13 MHz, CD_2_Cl_2_): δ 7.31, 7.26 (2s, br, 2 × 1H, C_6_H_2_), 4.78 (s, 5H, Cp), 4.16, 4.05 (2m, 2 × 1H, OCH_2_), 1.82 (d, *J*_HP_ = 2.9, 1H, CH), 1.64, 1.51, 1.31 (3s, 3 × 9H, ^*t*^Bu), 1.19 (t, *J*_HH_ = 7.1, 3H, CH_3_). ^13^C{^1^H} NMR (100.63 MHz, CD_2_Cl_2_): δ 237.3 (s, MoCO), 234.7 (d, *J*_CP_ = 6, MoCO), 194.7 (d, *J*_CP_ = 18, *C*O_2_Et), 160.0 [s, C^4^(C_6_H_2_)], 159.5 [d, *J*_CP_ = 8, C^2,6^(C_6_H_2_)], 150.8 [d, *J*_CP_ = 3, C^6,2^(C_6_H_2_)], 132.7 [s, C^1^(C_6_H_2_)], 123.8 [d, *J*_CP_ = 8, C^3,5^(C_6_H_2_)], 120.8 [d, *J*_CP_ = 11, C^5,3^(C_6_H_2_)], 91.2 (s, Cp), 63.4 (s, OCH_2_), 40.7, 39.4, 35.0 [3s, C^1^(^*t*^Bu)], 34.3 [s, C^2^(^*t*^Bu)], 33.4 [d, *J*_CP_ = 3, C^2^(^*t*^Bu)], 31.7 [s, C^2^(^*t*^Bu)], 24.5 (d, *J*_CP_ = 16, CH), 14.8 (s, CH_3_) ppm. The resonances for Mn-bound carbonyls could not be identified in this spectrum due to broadening induced by the quadrupolar ^55^Mn nucleus.

#### Preparation of [MoReCp(μ-η_^2^P,N_:κ_^2^P,N′_-PR*N_3_Bn)(CO)_6_] (**3a.1**)

Benzyl azide (12 μL, 0.095 mmol) was added to a toluene solution (10 mL) of compound **1a** (0.040 g, 0.051 mmol), and the mixture was stirred at room temperature for 3 h to give a dark green solution. Workup as for **2a** (elution with petroleum ether) gave minor orange and yellow fractions containing respectively unidentified species and the hydride complex [MoReCp(μ-H){μ-P(CH_2_CMe_2_)C_6_H_2_^*t*^Bu_2_}(CO)_6_].^6^ Elution with dichloromethane/petroleum ether (1/6) gave a green fraction, yielding compound **3a.1** as a green microcrystalline solid (0.035 g, 74%). Anal. calcd for C_36_H_41_MoN_3_O_6_PRe: C, 46.75; H, 4,47; N, 4.54. Found: C, 46.86; H, 4.28; N, 4.04. ^1^H NMR (400.13 MHz, CD_2_Cl_2_): δ 7.47–7.36 (m, 5H, Ph), 7.30, 7.25 (2s, br, 2 × 1H, C_6_H_2_), 5.81 (d, *J*_HH_ = 13.0, 1H, CH_2_), 5.14 (s, 5H, Cp), 5.13 (d, *J*_HH_ = 13.0, 1H, CH_2_), 1.80, 1.30, 1.26 (3s, 3 × 9H, ^*t*^Bu). ^13^C{^1^H} NMR (100.63 MHz, CD_2_Cl_2_): δ 255.8 (d, *J*_CP_ = 23, MoCO), 247.0 (s, MoCO), 190.0 (d, *J*_CP_ = 4, ReCO), 186.6 (d, *J*_CP_ = 10, ReCO), 185.2 (d, *J*_CP_ = 52, ReCO), 180.9 (d, *J*_CP_ = 6, ReCO), 156.9 [d, *J*_CP_ = 17, C^2,6^(C_6_H_2_)], 153.0 [d, *J*_CP_ = 5, C^6,2^(C_6_H_2_)], 148.9 [d, *J*_CP_ = 4, C^4^(C_6_H_2_)], 147.2 [d, *J*_CP_ = 28, C^1^(C_6_H_2_)], 135.9 [s, C^1^(Ph)], 130.1 [s, C^2^(Ph)], 129.1 [s, C^3^(Ph)], 128.7 [s, C^4^(Ph)], 125.3 [d, *J*_CP_ = 6, C^3,5^(C_6_H_2_)], 121.8 [d, *J*_CP_ = 11, C^5,3^(C_6_H_2_)], 94.0 (s, Cp), 73.0 (s, CH_2_), 40.3, 39.8, 35.0 [3s, C^1^(^*t*^Bu)], 34.8 [d, *J*_CP_ = 6, C^2^(^*t*^Bu)], 34.7, 30.9 [2s, C^2^(^*t*^Bu)].

#### Preparation of [MoReCp{μ-η^2^_P,N_:κ_^2^P,N′_-PR*N_3_(*p*-tol)}(CO)_6_] (**3a.2**)

A solution of *p*-tolyl azide (53 μL of a 0.5 M solution in ^*t*^BuOMe, 0.027 mmol) was added to a toluene solution (6 mL) of compound **1a** (0.020 g, 0.025 mmol), and the mixture was stirred at room temperature for 50 min to give a dark green solution. Workup as for **2a** gave first a minor yellow fraction containing the hydride complex [MoReCp(μ-H){μ-P(CH_2_CMe_2_)C_6_H_2_^*t*^Bu_2_}(CO)_6_].^5^ Elution with a 1/4 mixture gave a green fraction, yielding compound **3a.2** as a green microcrystalline solid (0.018 g, 78%). Crystals of **3a.2** were grown from a dichloromethane/petroleum solution at 253 K. Anal. calcd for C_36_H_41_MoN_3_O_6_PRe: C, 46.75; H, 4.47; N, 4.54. Found: C, 47.08; H, 5.09; N, 4.39. ^1^H NMR (400.13 MHz, CD_2_Cl_2_): δ 7.36 (d, *J*_HH_ = 2.2, 1H, C_6_H_2_), 7.31 (dd, *J*_HP_ = 4.2, *J*_HH_ = 2.2, 1H, C_6_H_2_), 7.25 [false d, *J*_HH_ = 8.2, 2H, H^2^(C_6_H_4_)], 7.18 [false d, *J*_HH_ = 8.2, 2H, H^3^(C_6_H_4_)], 5.16 (s, 5H, Cp), 2.40 (s, 3H, Me), 1.86, 1.38, 1.32 (3s, 3 × 9H, ^*t*^Bu). ^13^C{^1^H} NMR (100.63 MHz, CD_2_Cl_2_): δ 255.6 (d, *J*_CP_ = 23, MoCO), 247.8 (s, MoCO), 190.1 (s, ReCO), 187.8 (d, *J*_CP_ = 10, ReCO), 184.3 (d, *J*_CP_ = 49, ReCO), 180.9 (s, ReCO), 156.9 [d, *J*_CP_ = 17, C^2,6^(C_6_H_2_)], 153.2 [d, *J*_CP_ = 10, C^6,2^(C_6_H_2_)], 153.1 [s, C^4^(C_6_H_2_)], 149.1 [d, *J*_CP_ = 3, C^1^(C_6_H_4_)], 147.0 [d, *J*_CP_ = 27, C^1^(C_6_H_2_)], 138.1 [s, C^4^(C_6_H_4_)], 129.9 [s, C^2^(C_6_H_4_)], 125.6 [d, *J*_CP_ = 6, C^3,5^(C_6_H_2_)], 123.1 [s, C^3^(C_6_H_4_)], 121.8 [d, *J*_CP_ = 11, C^5,3^(C_6_H_2_)], 94.2 (s, Cp), 40.5, 39.8, 35.1 [3s, C^1^(^*t*^Bu)], 34.8 [d, *J*_CP_ = 5, C^2^(^*t*^Bu)], 34.5, 30.9 [2s, C^2^(^*t*^Bu)], 21.2 (s, Me).

#### Preparation of [MoMnCp(μ-η_^2^P,N_:κ^2^_P,N′_-PR*N_3_Bn)(CO)_6_] (**3b.1**)

Benzyl azide (6 μL, 0.048 mmol) was added to a toluene solution (8 mL) of compound **1b** (0.030 g, 0.045 mmol), and the mixture was stirred at 273 K for 1 h to give a dark green solution. The solvent was then removed under vacuum at 273 K, and the residue was processed, as described for **2a** (elution with a 1/8 mixture), to give first a minor fraction of unreacted **1b**. Elution with a 1/6 mixture gave a green fraction, yielding compound **3b.1** as a green microcrystalline solid (0.020 g, 56%). Elution with a 3/1 mixture gave a minor fraction of compound **5.1** (see below). Compound **3b.1** decomposed at room temperature in solution and in the solid state, and no microanalytical data were obtained for it. ^1^H NMR (400.13 MHz, CD_2_Cl_2_, 253 K): δ 7.43 (s, br, 2H, C_6_H_2_), 7.39 (m, 3H, Ph), 7.25 (m, 2H, Ph), 5.66 (d, *J*_HH_ = 13.2, 1H, CH_2_), 5.28 (s, 5H, Cp), 4.87 (d, *J*_HH_ = 13.2, 1H, CH_2_), 1.78 (s, 9H, *p*-^*t*^Bu), 1.27 (s, 18H, *o-*^*t*^Bu). ^13^C{^1^H} NMR (100.63 MHz, CD_2_Cl_2_, 253 K): δ 255.8 (d, *J*_CP_ = 23, MoCO), 246.7 (s, MoCO), 155.1 [d, *J*_CP_ = 18, C^2,6^(C_6_H_2_)], 152.4 [d, *J*_CP_ = 5, C^6,2^(C_6_H_2_)], 149.1 [d, *J*_CP_ = 3, C^4^(C_6_H_2_)], 147.7 [d, *J*_CP_ = 42, C^1^(C_6_H_2_)], 135.4 [s, C^1^(Ph)], 129.9 [s, C^2^(Ph)], 129.0 [s, C^3^(Ph)], 128.6 [s, C^4^(Ph)], 124.8 [d, *J*_CP_ = 5, C^3,5^(C_6_H_2_)], 121.7 [d, *J*_CP_ = 11, C^5,3^(C_6_H_2_)], 94.2 (s, Cp), 72.0 (s, CH_2_), 39.8, 39.5, 35.0 [3s, C^1^(^*t*^Bu)], 34.5 [d, *J*_CP_ = 5, C^2^(^*t*^Bu)], 34.4, 30.8 [2s, C^2^(^*t*^Bu)]. Resonances for Mn-bound carbonyls could not be identified in this spectrum due to broadening induced by the quadrupolar ^55^Mn nucleus.

### Low-Temperature Reaction of 1b with (*p*-tol)N_3_

A solution of *p*-tolyl azide (90 μL of a 0.5 M solution in ^*t*^BuOMe, 0.045 mmol) was added to a toluene solution (8 mL) of compound **1b** (0.030 g, 0.045 mmol), and the mixture was stirred at 273 K for 90 min to give a dark green solution. Workup as for **3b.1** gave a minor fraction of unreacted **1b**. Elution with a 1/6 mixture gave a minor orange fraction of compound [MoMnCp{μ-κ_P_:κ_N_-PR*N(*p*-tol)}(CO)_6_] (**4b**), then a major green fraction, yielding compound [MoReCp{μ-η_^2^P,N_:κ_^2^P,N′_-PR*N_3_(*p*-tol)}(CO)_6_] (**3b.2**) as a green microcrystalline solid (0.020 g, 56%). Elution with a 1/3 mixture gave a minor orange fraction of compound **5.2** (see below). Compound **3b.2** decomposed at room temperature in solution and in the solid state, and no microanalytical data were obtained for it. *Spectroscopic data for**3b.2***: ^1^H NMR (400.13 MHz, CD_2_Cl_2_, 253 K): δ 7.37, 7.29 (2s, 2 × 1H, C_6_H_2_), 7.26 [false d, *J*_HH_ = 7.9, 2H, H^2^(C_6_H_4_)], 7.18 [false d, *J*_HH_ = 7.9, 2H, H^3^(C_6_H_4_)], 5.21 (s, 5H, Cp), 2.40 (s, 3H, Me), 1.82, 1.38, 1.32 (3s, 3 × 9H, ^*t*^Bu). ^13^C{^1^H} NMR (100.63 MHz, CD_2_Cl_2_, 253 K): δ 255.8 (s, MoCO), 248.0 (s, MoCO), 155.7 [d, *J*_CP_ = 17, C^2,6^(C_6_H_2_)], 153.1 [s, C^4^(C_6_H_2_)], 152.8 [d, *J*_CP_ = 5, C^6,2^(C_6_H_2_)], 148.9 [d, *J*_CP_ = 3, C^1^(C_6_H_4_)], 147.9 [d, *J*_CP_ = 40, C^1^(C_6_H_2_)], 137.7 [s, C^4^(C_6_H_4_)], 129.7 [s, C^2^(C_6_H_4_)], 125.2 [d, *J*_CP_ = 5, C^3,5^(C_6_H_2_)], 122.6 [s, C^3^(C_6_H_4_)], 121.1 [d, *J*_CP_ = 10, C^5,3^(C_6_H_2_)], 93.9 (s, Cp), 40.0, 39.3, 34.8 [3s, C^1^(^*t*^Bu)], 34.3 [d, *J*_CP_ = 5, C^2^(^*t*^Bu)], 33.9, 30.6 [2s, C^2^(^*t*^Bu)], 21.1 (s, Me). The resonances for Mn-bound carbonyls could not be identified in this spectrum due to broadening induced by the quadrupolar ^55^Mn nucleus. *Spectroscopic data for**4b***: ^1^H NMR (400.13 MHz, CD_2_Cl_2_): δ 7.31 (s, br, 2H, C_6_H_2_), 6.79 [false d, *J*_HH_ = 8.1, 2H, H^2^(C_6_H_4_)], 6.53 [false d, *J*_HH_ = 8.1, 2H, H^3^(C_6_H_4_)], 5.38 (s, 5H, Cp), 2.15 (s, 3H, Me), 1.46 (s, br, 18H, *o*-^*t*^Bu), 1.26 (s, 9H, *p*-^*t*^Bu).

#### Preparation of [MoReCp{μ-κ_P_:κ_N_-PR*N(*p*-tol)}(CO)_6_] (**4a**)

A toluene solution (6 mL) of compound **3a.2** (0.015 g, 0.016 mmol) was stirred at 333 K for 3 h to give an orange solution. Workup as for **2a** (elution with a 1/20 mixture) gave an orange fraction, yielding compound **4a** as an orange microcrystalline solid (0.010 g, 70%). Crystals of **4a** were grown from a concentrated petroleum ether solution at 253 K. Anal. calcd for C_36_H_41_MoNO_6_PRe: C, 48.21; H, 4.61; N, 1.56. Found: C, 48.47; H, 4.47; N, 1.64. ^1^H NMR (300.13 MHz, CD_2_Cl_2_): δ 7.35 (d, *J*_HH_ = 2.6, 2H, C_6_H_2_), 6.81 [false d, *J*_HH_ = 8.3, 2H, H^2^(C_6_H_4_)], 6.49 [false d, *J*_HH_ = 8.3, 2H, H^3^(C_6_H_4_)], 5.37 (s, 5H, Cp), 2.16 (s, 3H, Me), 1.45 (s, 18H, *o*-^*t*^Bu), 1.28 (s, 9H, *p*-^*t*^Bu). ^13^C{^1^H} NMR (100.63 MHz, CD_2_Cl_2_): δ 230.7 (d, *J*_CP_ = 14, 2MoCO), 197.9 (s, br, 2ReCO), 191.4 (s, ReCO), 187.4 (s, ReCO), 152.6 [s, C^4^(C_6_H_2_)], 150.2 [d, *J*_CP_ = 5, C^2^(C_6_H_2_)], 149.9 [d, *J*_CP_ = 18, C^1^(C_6_H_4_)], 141.1 [d, *J*_CP_ = 30, C^1^(C_6_H_2_)], 134.0 [s, C^4^(C_6_H_4_)], 128.9 [s, C^3^(C_6_H_4_)], 125.4 [d, *J*_CP_ = 6, C^2^(C_6_H_4_)], 123.3 [d, *J*_CP_ = 10, C^3^(C_6_H_2_)], 90.7 (s, Cp), 39.4 [s, C^1^(*o*-^*t*^Bu)], 35.3 [s, C^1^(*p*-^*t*^Bu)], 34.3 [s, C^2^(*o*-^*t*^Bu)], 31.1 [s, C^2^(*p*-^*t*^Bu)], 20.6 (s, Me).

#### Preparation of [Mn(κ_^2^P,N_-PR*NHNNBn)(CO)_3_] (**5.1**)

A toluene solution (6 mL) of compound **3b.1** (0.020 g, 0.025 mmol) was stirred at room temperature for 4 h to give an orange solution. Workup as for **2a** (elution with a 1/6 mixture) gave an orange fraction, yielding compound **5.1** as an orange microcrystalline solid (0.008 g, 58%). Crystals of **5.1** were grown from a toluene/petroleum solution at 253 K. Anal. calcd for C_28_H_37_MnN_3_O_3_P: C, 61.20; H, 6.79; N, 7.65. Found: C, 60.89; H, 6.24; N, 7.02. IR data (Nujol): ν(NH) 3329(m); ν(CO) 1999 (vs), 1922 (s), 1906 (m). ^1^H NMR (400.13 MHz, CD_2_Cl_2_): δ 10.56 (s, br, 1H, NH), 7.59 (d, *J*_HH_ = 3.1, 2H, C_6_H_2_), 7.36–7.28 (m, 5H, Ph), 6.17 (s, 2H, CH_2_), 1.46 (s, 18H, *o*-^*t*^Bu), 1.38 (s, 9H, *p*-^*t*^Bu). ^13^C{^1^H} NMR (100.63 MHz, CD_2_Cl_2_): δ 226.9 (s, br, 3MnCO), 159.4 [s, C^2^(C_6_H_2_)], 155.3 [s, C^4^(C_6_H_2_)], 140.2 [s, C^1^(C_6_H_2_)], 128.9 [s, C^2^(Ph)], 128.6 [s, C^3^(Ph)], 128.1 [s, C^4^(Ph)], 125.9 [s, br, C^1^(Ph)], 123.1 [d, *J*_CP_ = 7, C^3^(C_6_H_2_)], 74.7 (s, CH_2_), 38.9 [s, C^1^(*o*-^*t*^Bu)], 35.9 [s, C^1^(*p*-^*t*^Bu)], 33.8 [s, C^2^(*o*-^*t*^Bu)], 31.3 [s, C^2^(*p*-^*t*^Bu)].

#### Preparation of [Mn{κ^2^_P,N_-PR*NHNN(*p*-tol)}(CO)_3_] (**5.2**)

A toluene solution (6 mL) of compound **3b.2** (0.020 g, 0.025 mmol) was stirred at room temperature for 6 h, to give an orange solution. Workup as for **2a** (elution with a 1/8 mixture) gave a minor orange fraction of **4b**, then a minor yellow fraction containing unidentified species. Elution with a 2/1 mixture gave a major orange fraction, yielding compound **5.2** as an orange microcrystalline solid (0.006 g, 44%). Anal. calcd for C_28_H_37_MnN_3_O_3_P: C, 61.20; H, 6.79; N, 7.65. Found: C, 60.80; H, 6.15; N, 6.93. ^1^H NMR (400.13 MHz, CD_2_Cl_2_): δ 10.89 (s, br, 1H, NH), 7.63 (d, *J*_HH_ = 3.1, 2H, C_6_H_2_), 7.54 [false d, *J*_HH_ = 8.1, 2H, H^2^(C_6_H_4_)], 7.32 [false d, *J*_HH_ = 8.1, 2H, H^3^(C_6_H_4_)], 2.46 (s, 3H, Me), 1.52 (s, 18H, *o*-^*t*^Bu), 1.39 (s, 9H, *p*-^*t*^Bu). ^13^C{^1^H} NMR (100.63 MHz, CD_2_Cl_2_, 253 K): δ 227.1 (s, br, 3MnCO), 159.2 [s, C^2^(C_6_H_2_)], 155.6 [s, C^1^(C_6_H_4_)], 155.2 [s, C^4^(C_6_H_2_)], 137.7 [s, C^4^(C_6_H_4_)], 129.5 [s, C^2^(C_6_H_4_)], 124.2 [s, C^3^(C_6_H_4_)], 123.1 [d, *J*_CP_ = 7, C^3^(C_6_H_2_)], 38.9 [s, C^1^(*o*-^*t*^Bu)], 35.9 [s, C^1^(*p*-^*t*^Bu)], 33.7 [s, C^2^(*o*-^*t*^Bu)], 31.2 [s, C^2^(*p*-^*t*^Bu)], 21.3 (s, Me); the resonance for the C^1^(C_6_H_2_) atom could not be located in the spectrum.

### X-ray Structure Determination of Compounds **2a** and **5.1**

Data collection for these compounds was performed at 100 K on a Bruker D8 Venture Photon III 14 κ-geometry diffractometer, using Mo Kα radiation. Structure solution and refinements were performed following general procedures described before^5,6^ to give the residuals shown in Table S1. In compound **2a**, two independent but otherwise similar molecules were present in the unit cell; both of them had a disordered ^*t*^Bu group, satisfactorily modeled over two sites with 0.70/0.30 and 0.75/0.25 occupancies, respectively. The disordered carbon atoms were refined isotropically, and this caused B-level alerts in the corresponding checkcif file. For compound **5.1**, there was a disordered ^*t*^Bu group too, satisfactorily modeled over two sites with 0.65/0.35 occupancies in this case. There was also a toluene molecule (one per two molecules of the complex) placed on an inversion center, which caused symmetry-imposed disorder (50% occupancies), which could be modeled while applying restraints on the C–C bond lengths and the ring planarity. The disordered carbon atoms were refined isotropically, and this again caused the appearance of B-level alerts in the corresponding checkcif file.

### X-ray Structure Determination of Compounds **3a.2** and **4a**

Data collection for these compounds was performed at ca. 150 K on an Oxford Diffraction Xcalibur Nova single-crystal diffractometer using Cu Kα radiation Structure solution, and refinements were performed following general procedures described before^5,6^ to give the residuals shown in Table S1.

## Supporting Information Available

Crystal data for compounds **2a**, **3a.2**, **4a**, and **5.1** (CCDC 2193261–2193264), and IR and NMR spectra for all new compounds (PDF)
