# Supplementary material for: P–C, P–N, and M–N Bond Formation Processes in Reactions of Heterometallic Phosphinidene-Bridged MoMn and MoRe Complexes with Diazoalkanes and Organic Azides to Build Three- to Five-Membered Phosphametallacycles
Source: Inorg Chem. 2022 Nov 9;61(46):18486–95. doi: 10.1021/acs.inorgchem.2c02720 (PMC9682477; doi:10.1021/acs.inorgchem.2c02720)
Supplement: Supplementary file 1 — ic2c02720_si_001.pdf [file ic2c02720_si_001.pdf]

Supporting Information

P–C, P–N and M–N Bond Formation Processes in Reactions of Heterometallic Phosphinidene-Bridged MoMn and MoRe Complexes with Diazoalkanes and Organic Azides to Build 3- to 5-Membered Phosphametallacycles.

M. Angeles Alvarez, Pablo M. Cuervo, M. Esther García, Miguel A. Ruiz,* and Patricia Vega

Departamento de Química Orgánica e Inorgánica/IUQOEM, Universidad de Oviedo, E-33071 Oviedo, Spain.

Corresponding Author E-mail: mara@uniovi.es.

Table S1. Crystal Data for New Compounds

	**2a**	**3a.2**	**4a**	**5.1**·1/2tol
mol formula	$C_{32}H_{40}MoO_7PRe$	$C_{36}H_{41}MoN_3O_6PRe$	$C_{36}H_{41}MoNO_6PRe$	$C_{63}H_{82}Mn_2N_6O_6P_2$
mol wt	849.76	924.83	896.81	1191.17
cryst syst	orthorhombic	monoclinic	monoclinic	monoclinic
space group	$P2_12_12_1$	$P2_1/c$	$P2_1/c$	$P2_1/c$
radiation (λ, Å)	0.71073	1.54184	1.54184	0.71073
a, Å	17.8289(6)	10.98090(10)	11.1447(1)	9.0539(3)
b, Å	18.0992(6)	16.7265(2)	18.3342(2)	17.9035(5)
c, Å	20.6892(8)	19.9432(2)	17.5003(2)	19.7277(6)
α, deg	90	90	90	90
β, deg	90	94.357(1)	94.044(1)	101.5070(10)
γ, deg	90	90	90	90
V, Å^3	6676.2(4)	3652.42(7)	3566.92(6)	3133.52(17)
Z	8	4	4	2
calcd density, g cm^{-3}	1.691	1.682	1.670	1.263
absorp coeff, mm^{-1}	4.095	9.997	10.198	0.507
temperature, K	100.0(1)	150(2)	150.0(1)	100.0(1)
θ range (deg)	2.25/36.38	3.45/69.56	3.50 /69.64	2.11 /26.37
index ranges (h, k, l)	–29, 29; –30, 29 –34, 34	–13, 9; –20, 18 –22, 24	–13, 13; –21, 22 –20, 21	–11, 11; –22, 22 –24, 24
no. of reflns collected	183640	20579	18090	151705
no. of indep reflns (R_{int})	32463 (0.0413)	6806 (0.0340)	6582 (0.0267)	6403 (0.0635)
reflns with $I > 2\sigma(I)$	29918	6464	6050	5532
R indexes [data with $I > 2\sigma(I)$]^a	R_1 = 0.0237 wR_2 = 0.0428^b	R_1 = 0.0286 wR_2 = 0.0736^c	R_1 = 0.0234 wR_2 = 0.0585^d	R_1 = 0.0409 wR_2 = 0.0995^e
R indexes (all data)^a	R_1 = 0.0292 wR_2 = 0.0444^b	R_1 = 0.0302 wR_2 = 0.0752^c	R_1 = 0.0262 wR_2 = 0.0604^d	R_1 = 0.0493 wR_2 = 0.1067^e
GOF	1.010	1.047	1.043	1.076
no. of restraints/params	0 / 752	0 / 443	0 / 415	0 / 341
$\Delta\rho$(max., min.), eÅ^{-3}	0.749 / –0.940	1.113 / –1.754	0432 / –1.119	0.916 / –0.428
CCDC deposition no	2193261	2193262	2193263	2193264

^{a} $R = \Sigma||F_o| - |F_c|| / \Sigma|F_o|$. $wR = [\Sigma w(|F_o|^2 - |F_c|^2)^2 / \Sigma w|F_o|^2]^{1/2}$. $w = 1/[\sigma^2(F_o^2) + (aP)^2 + bP]$ where $P = (F_o^2 + 2F_c^2)/3$. ^{b} a = 0.0150, b = 1.9637. ^{c} a = 0.0433, b = 2.4828. ^{d} a = 0.0323, b = 0.7140. ^{e} a = 0.0410, b = 3.7353.

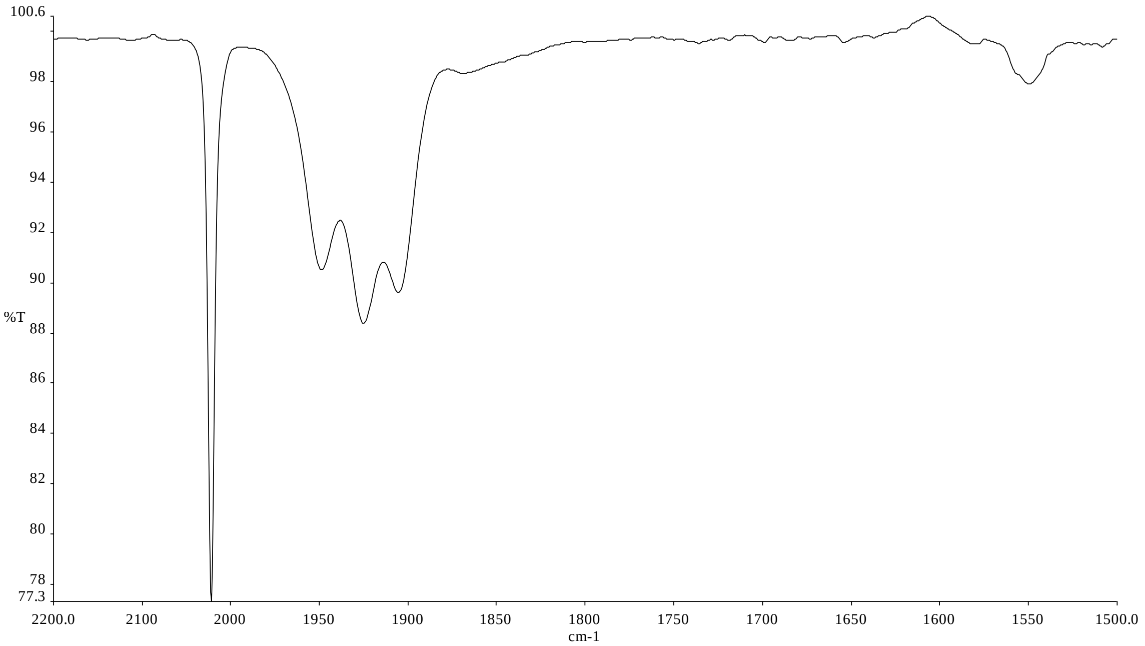

Figure S1. IR spectrum of compound **2a** in dichloromethane solution.

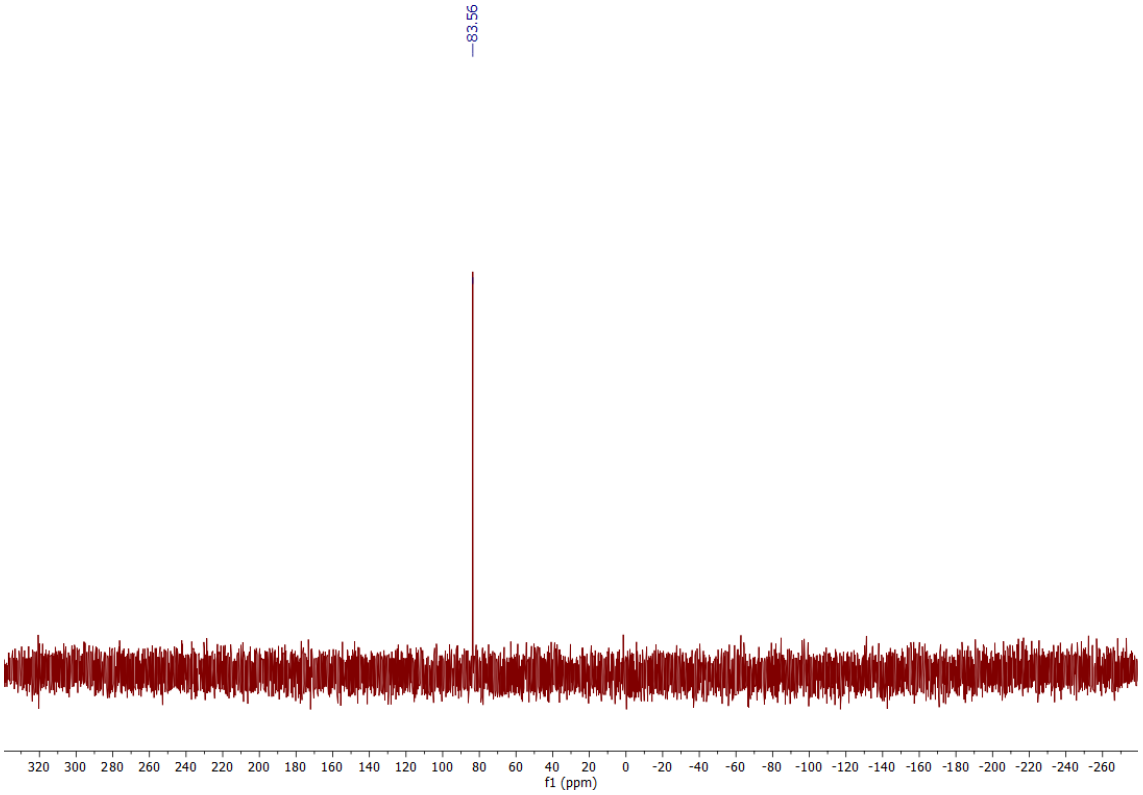

Figure S2. $^{31}P\{^{1}H\}$ NMR spectrum of compound **2a** (CD_2Cl_2).

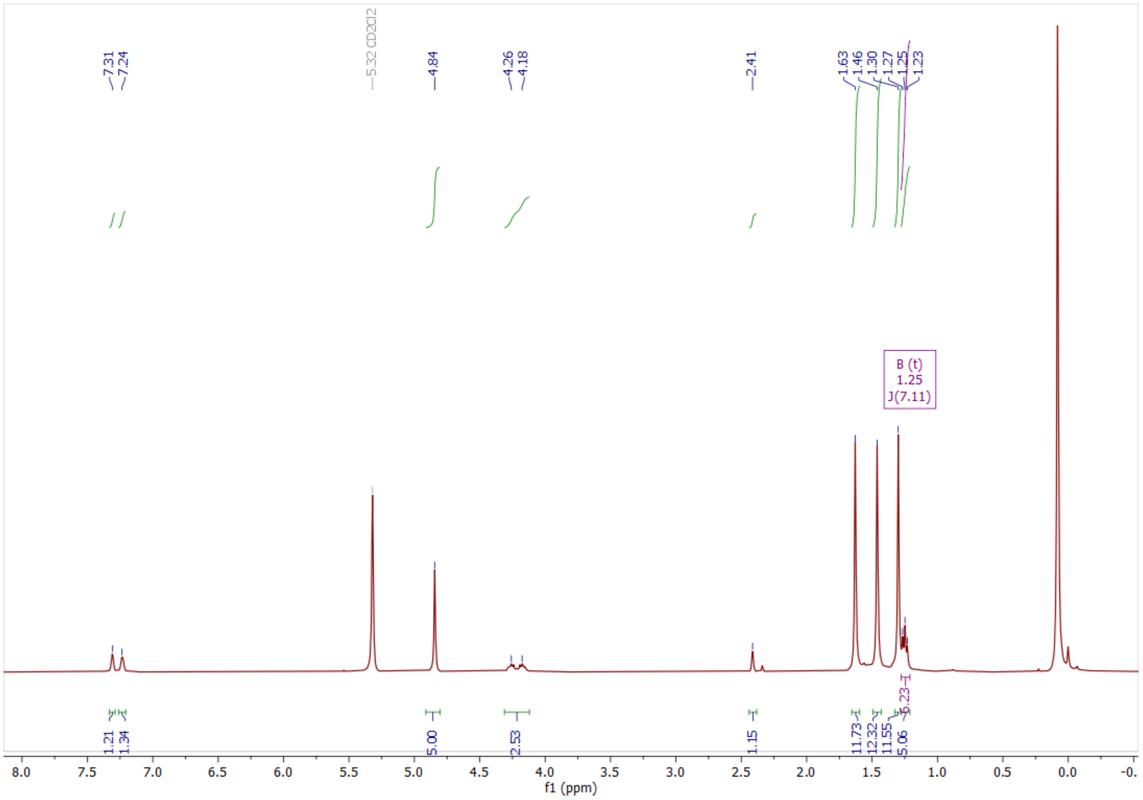

Figure S3. ^{1}H NMR spectrum of compound **2a** (CD_2Cl_2).

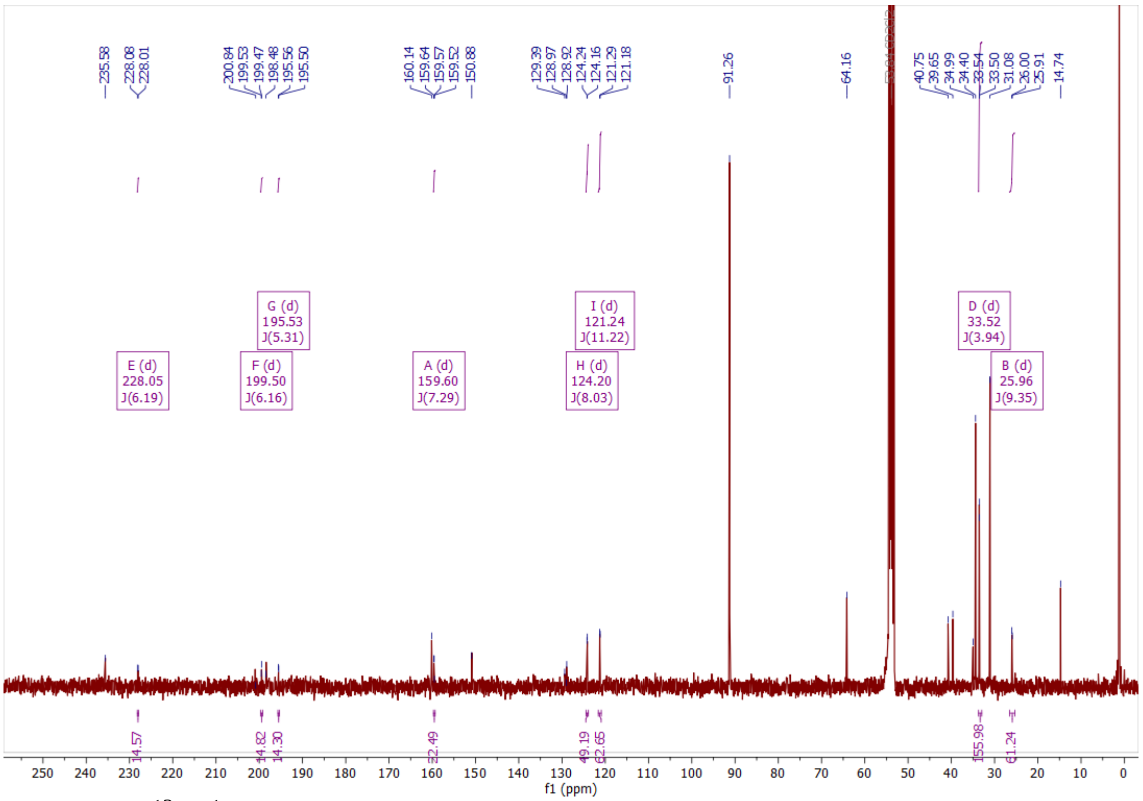

Figure S4. $^{13}C\{^{1}H\}$ NMR spectrum of compound **2a** (CD_2Cl_2).

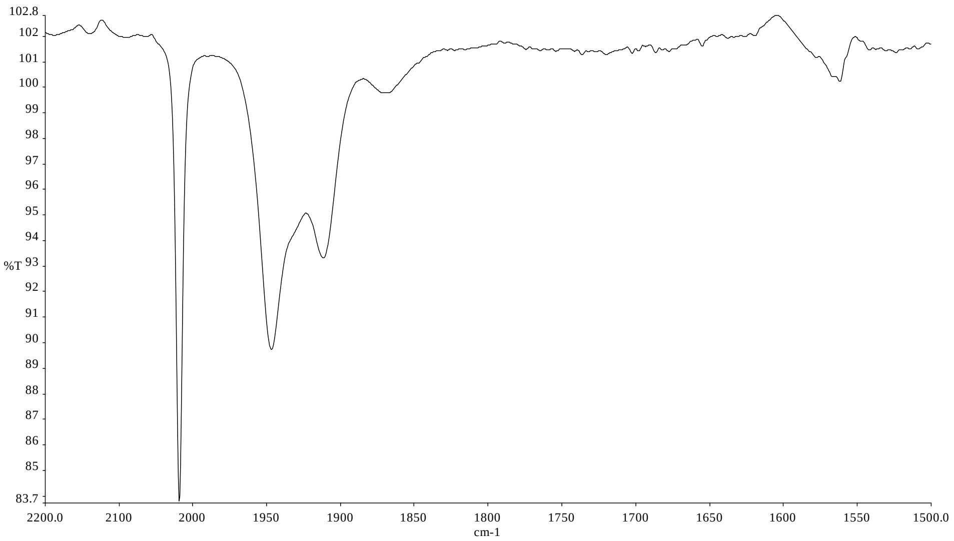

Figure S5. IR spectrum of compound **2b** in dichloromethane solution.

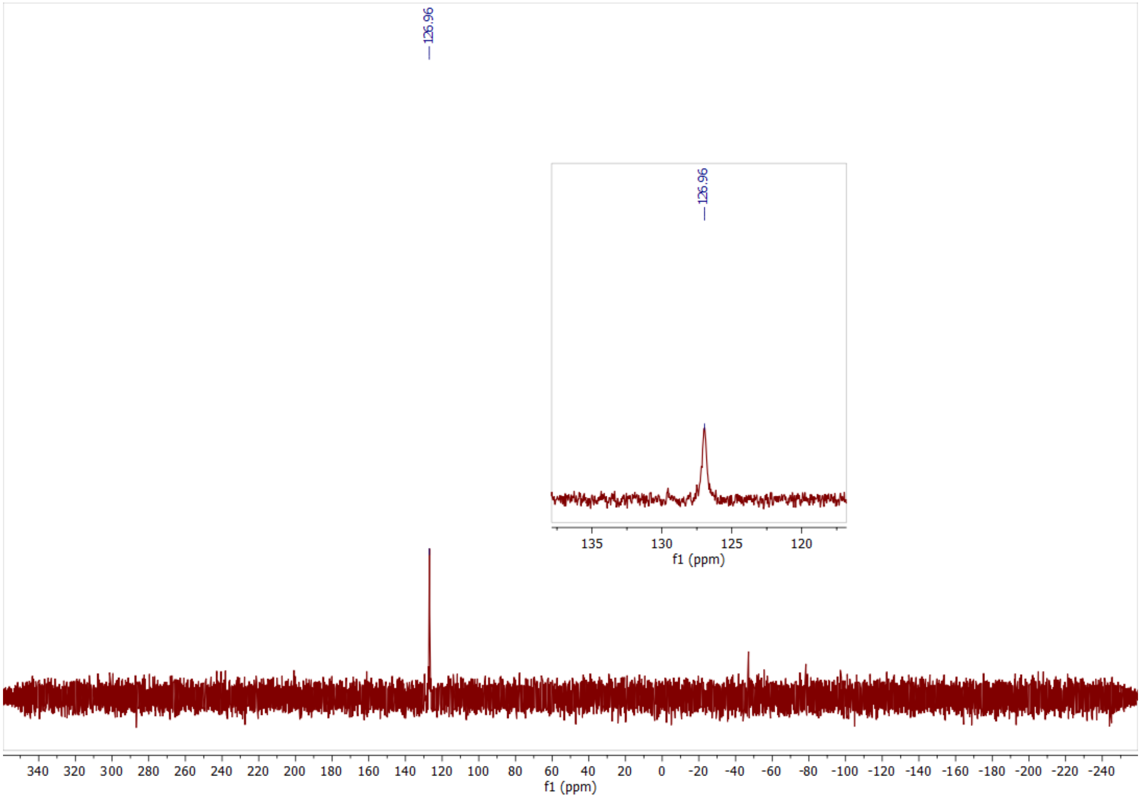

Figure S6. $^{31}P\{^1H\}$ NMR spectrum of compound **2b** (CD_2Cl_2).

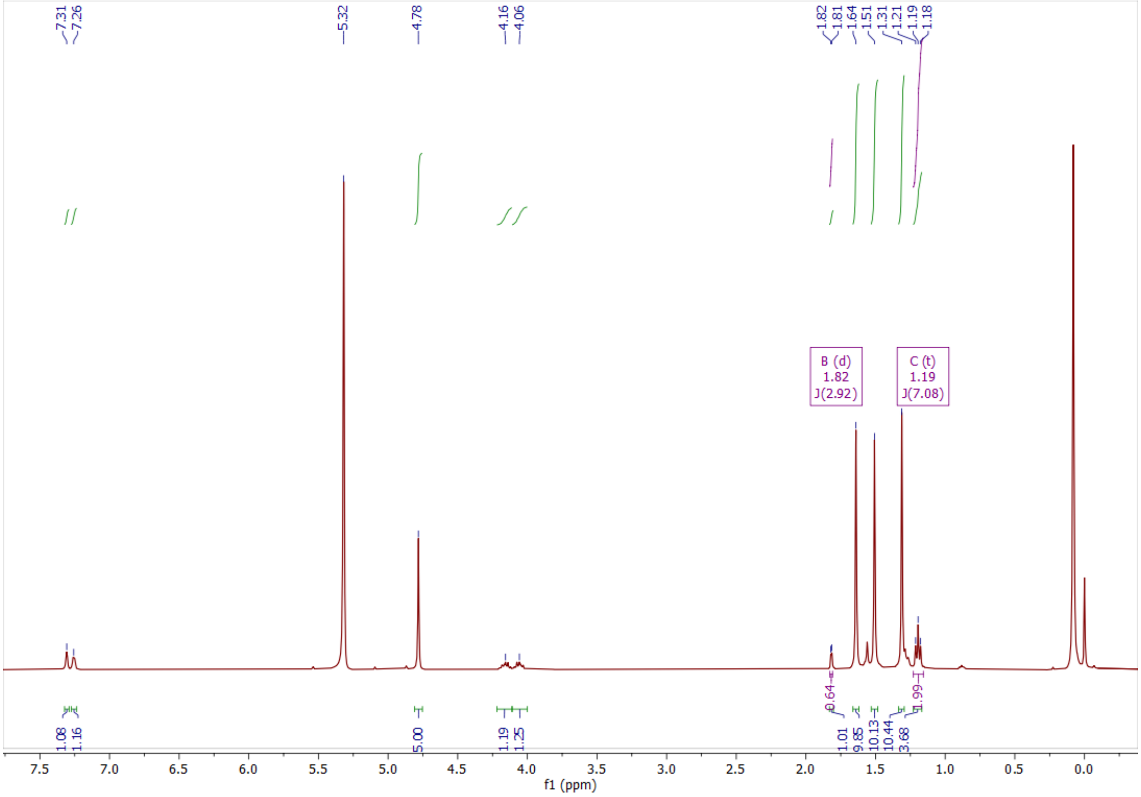

Figure S7. ^{1}H NMR spectrum of compound **2b** (CD_2Cl_2).

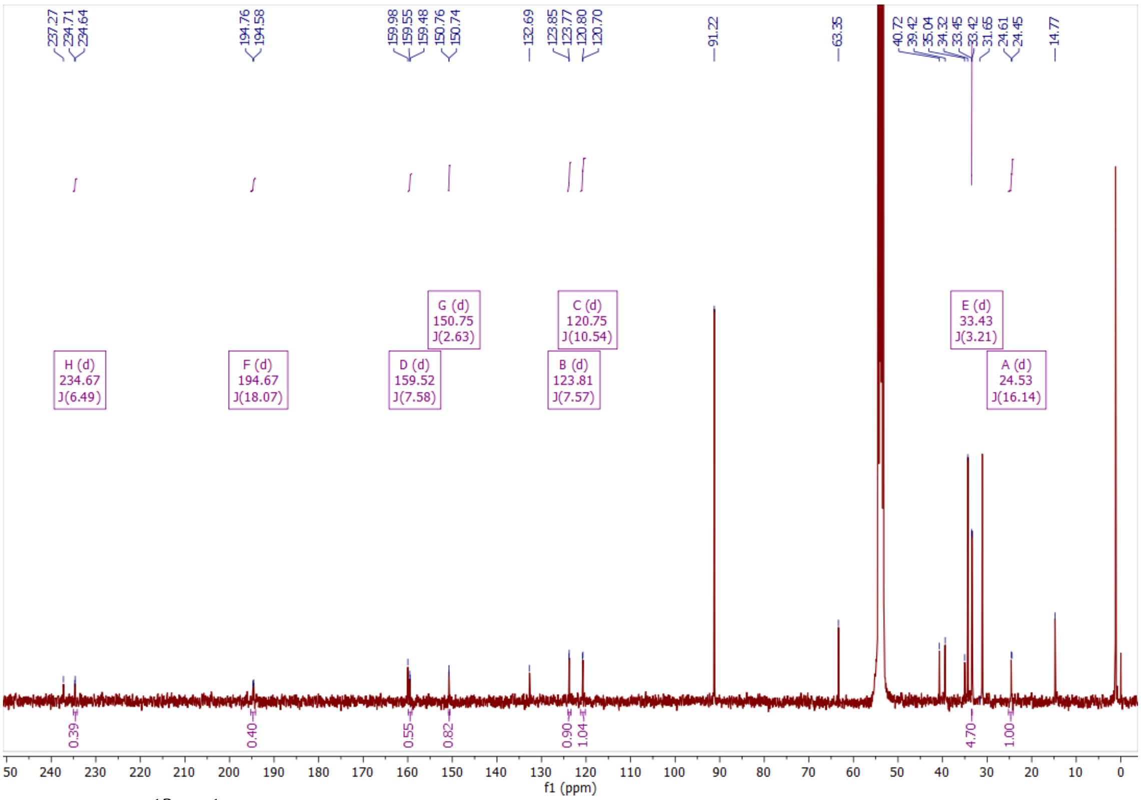

Figure S8. $^{13}C\{^{1}H\}$ NMR spectrum of compound **2b** (CD_2Cl_2).

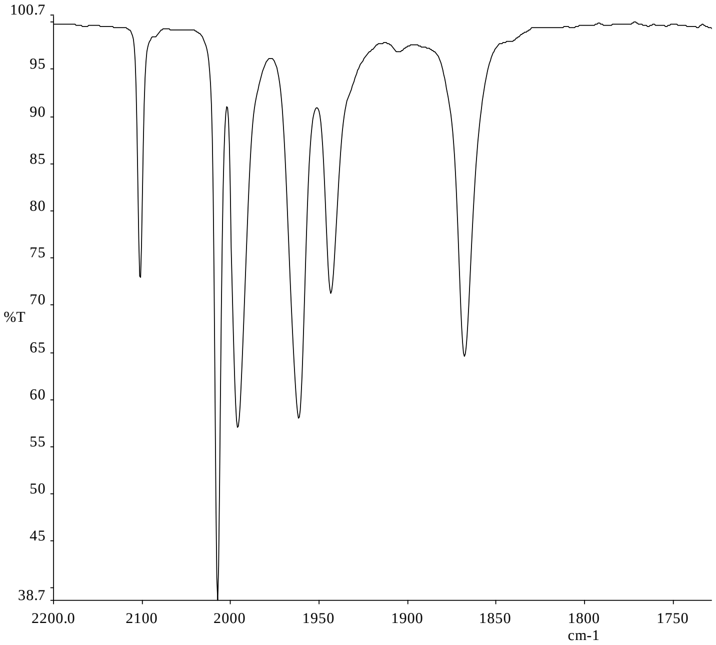

Figure S9. IR spectrum of compound **3a.1** in petroleum ether solution.

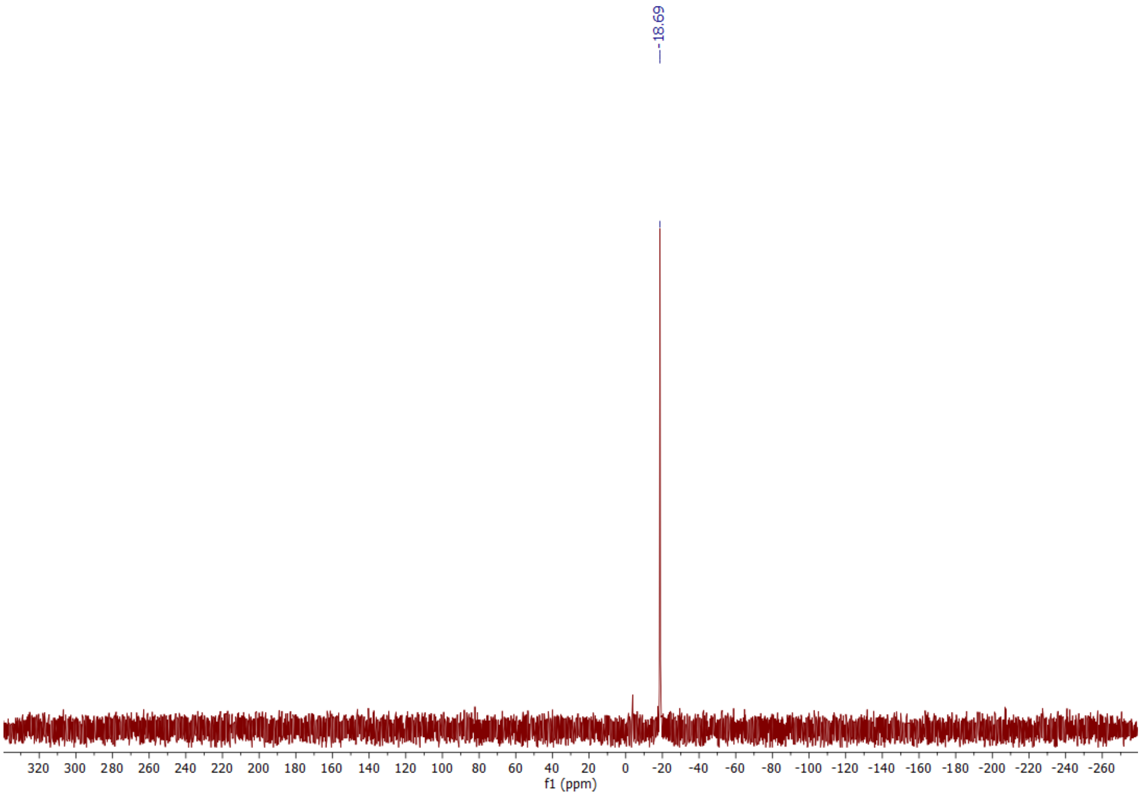

Figure S10. $^{31}P\{^{1}H\}$ NMR spectrum of compound **3a.1** (CD_2Cl_2).

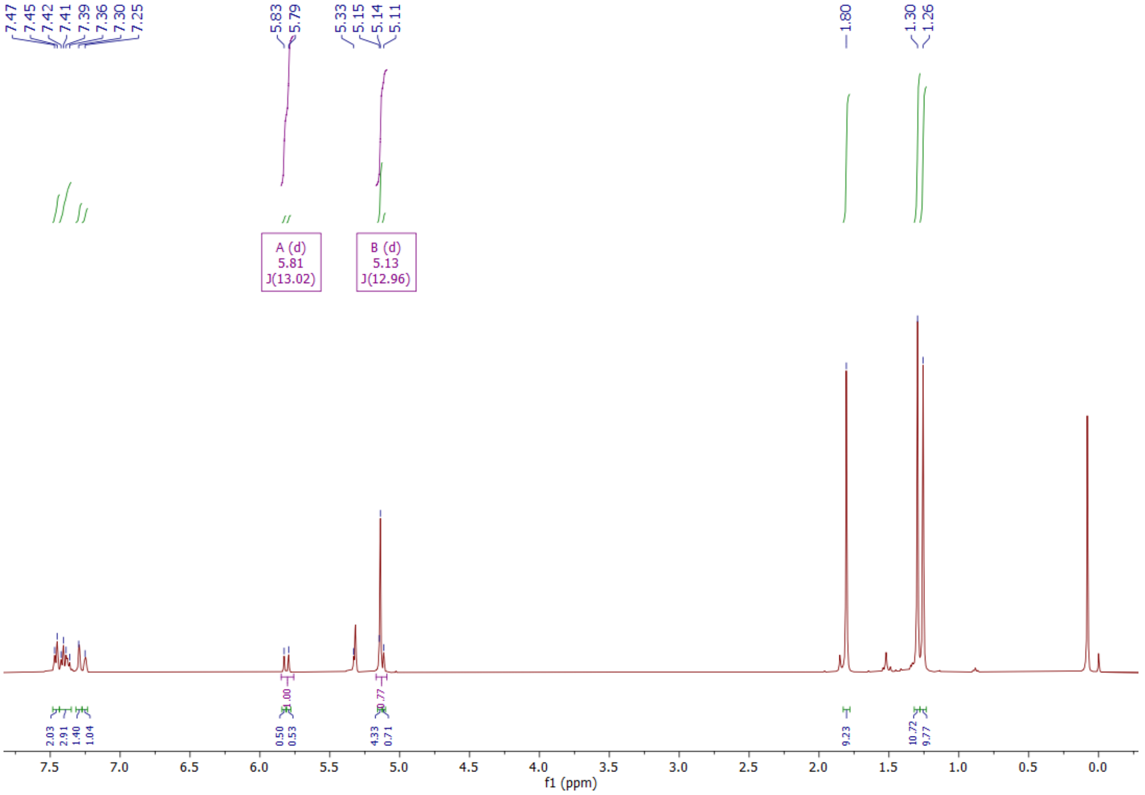

Figure S11. ^{1}H NMR spectrum of compound **3a.1** (CD_2Cl_2).

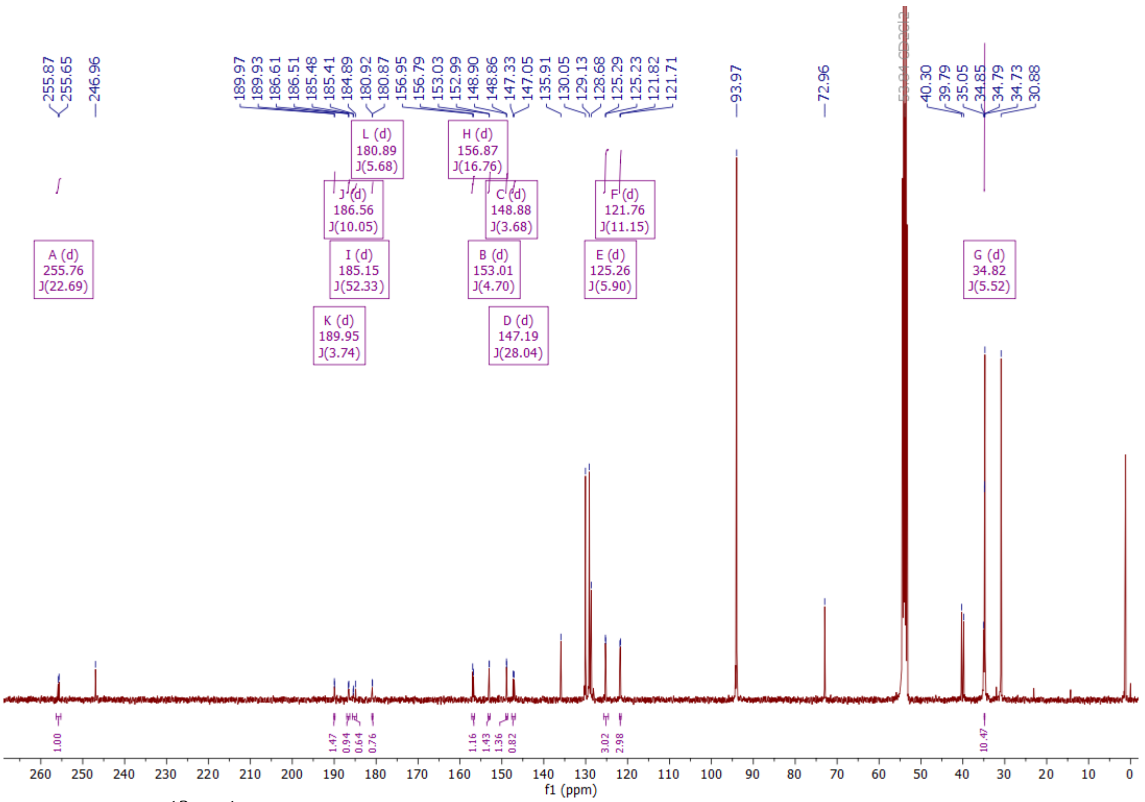

Figure S12. $^{13}C\{^{1}H\}$ NMR spectrum of compound **3a.1** (CD_2Cl_2).

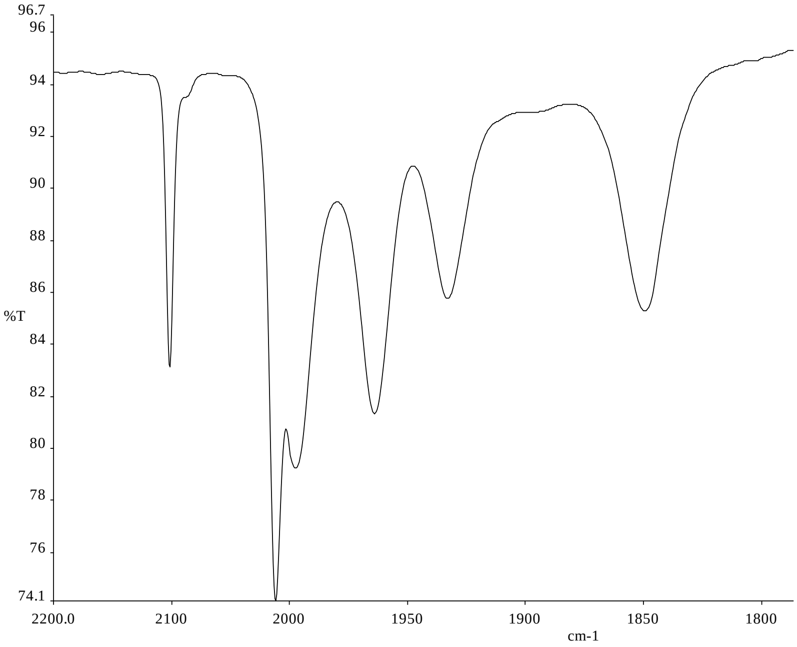

Figure S13. IR spectrum of compound **3a.2** in dichloromethane solution.

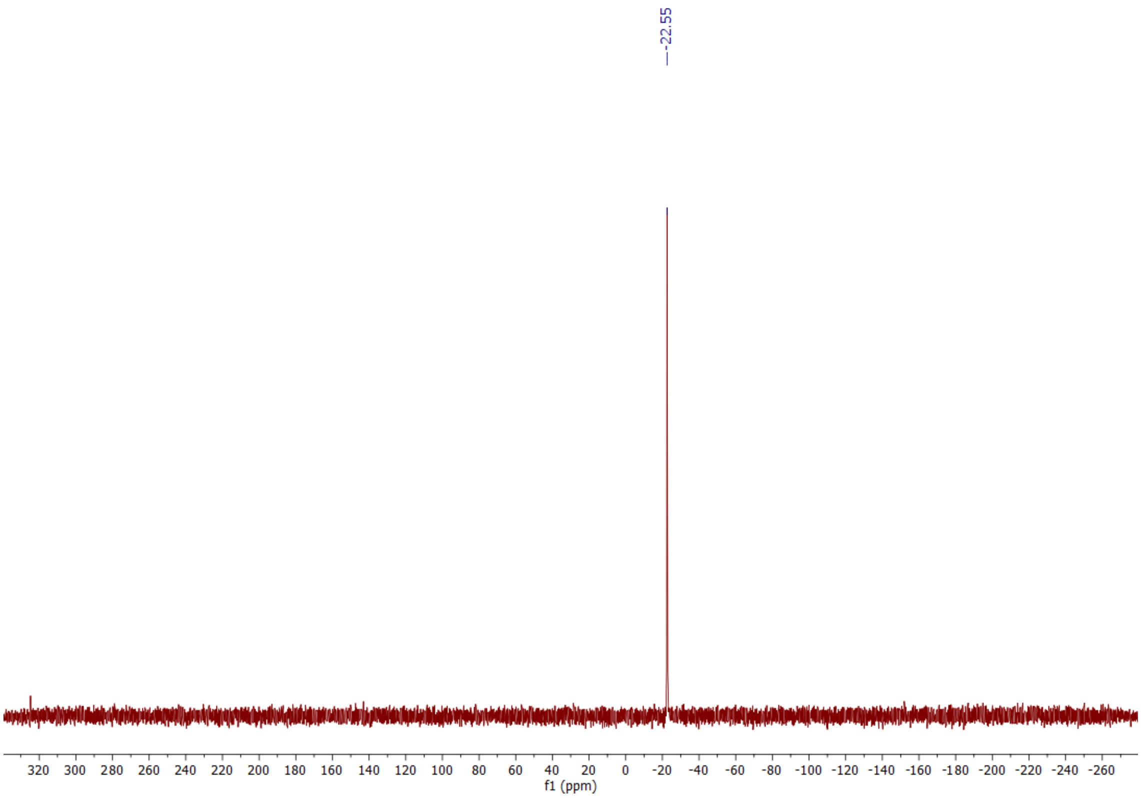

Figure S14. $^{31}P\{^1H\}$ NMR spectrum of compound **3a.2** (CD_2Cl_2).

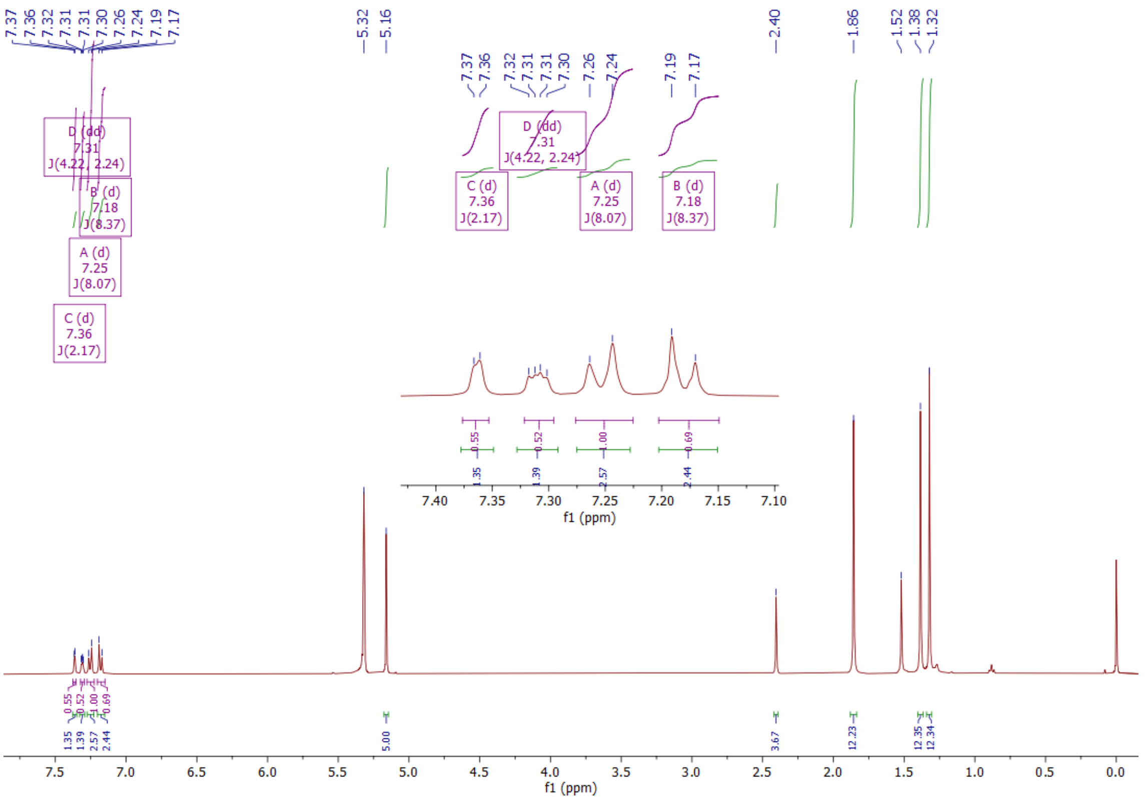

Figure S15. ^{1}H NMR spectrum of compound **3a.2** (CD_2Cl_2).

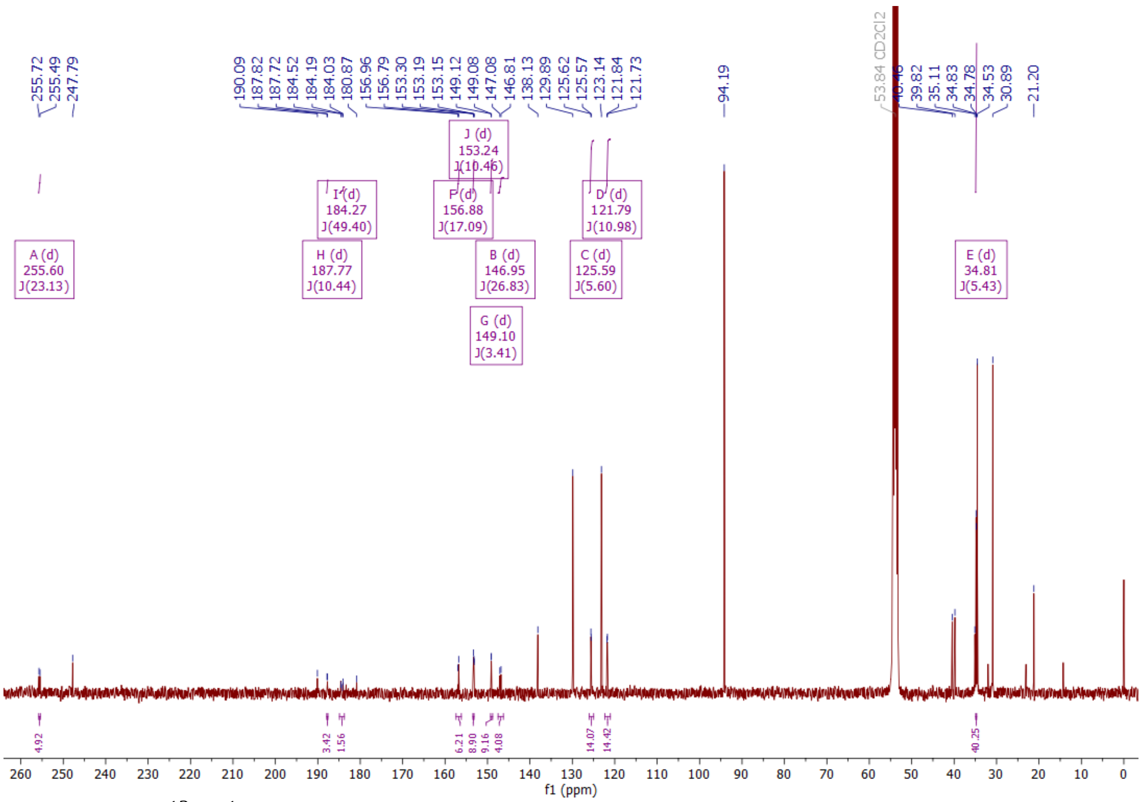

Figure S16. $^{13}C\{^{1}H\}$ NMR spectrum of compound **3a.2** (CD_2Cl_2).

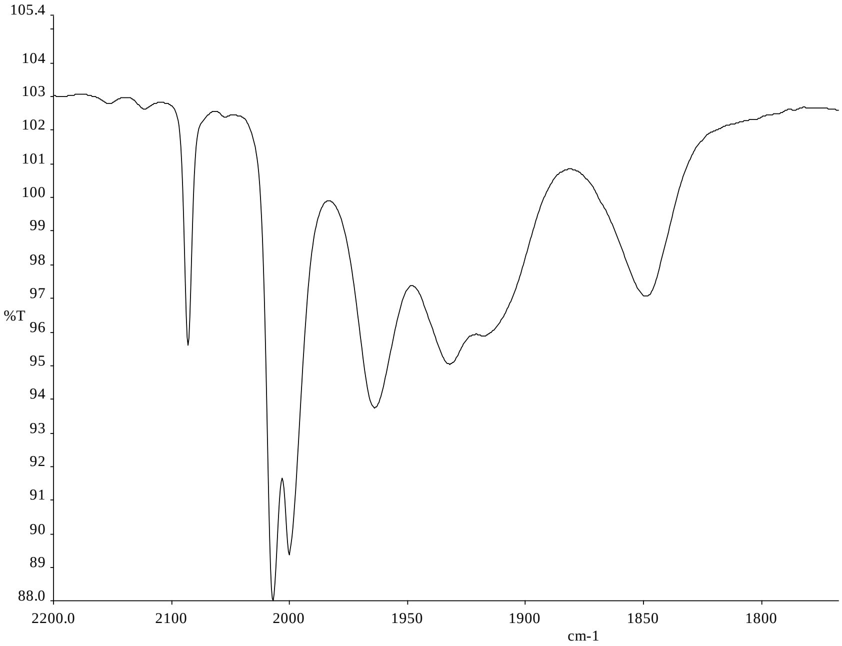

Figure S17. IR spectrum of compound **3b.1** in dichloromethane solution.

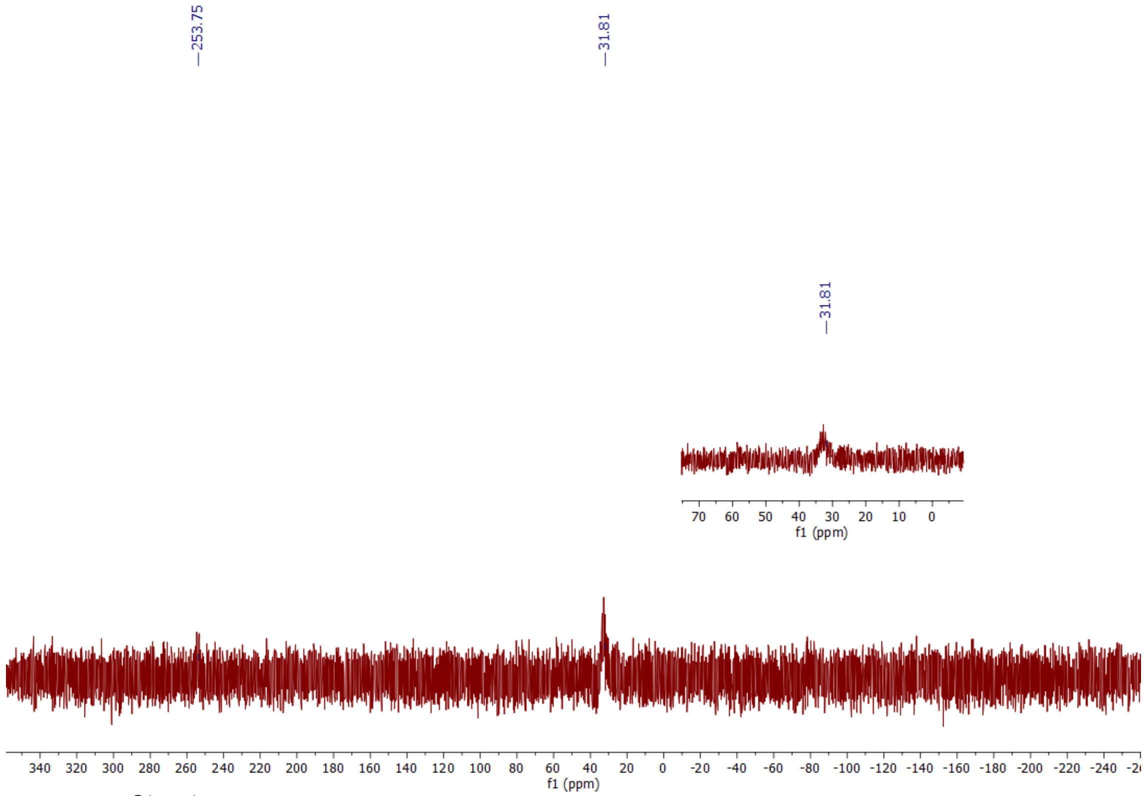

Figure S18. $^{31}P\{^{1}H\}$ NMR spectrum of compound **3b.1** (CD_2Cl_2).

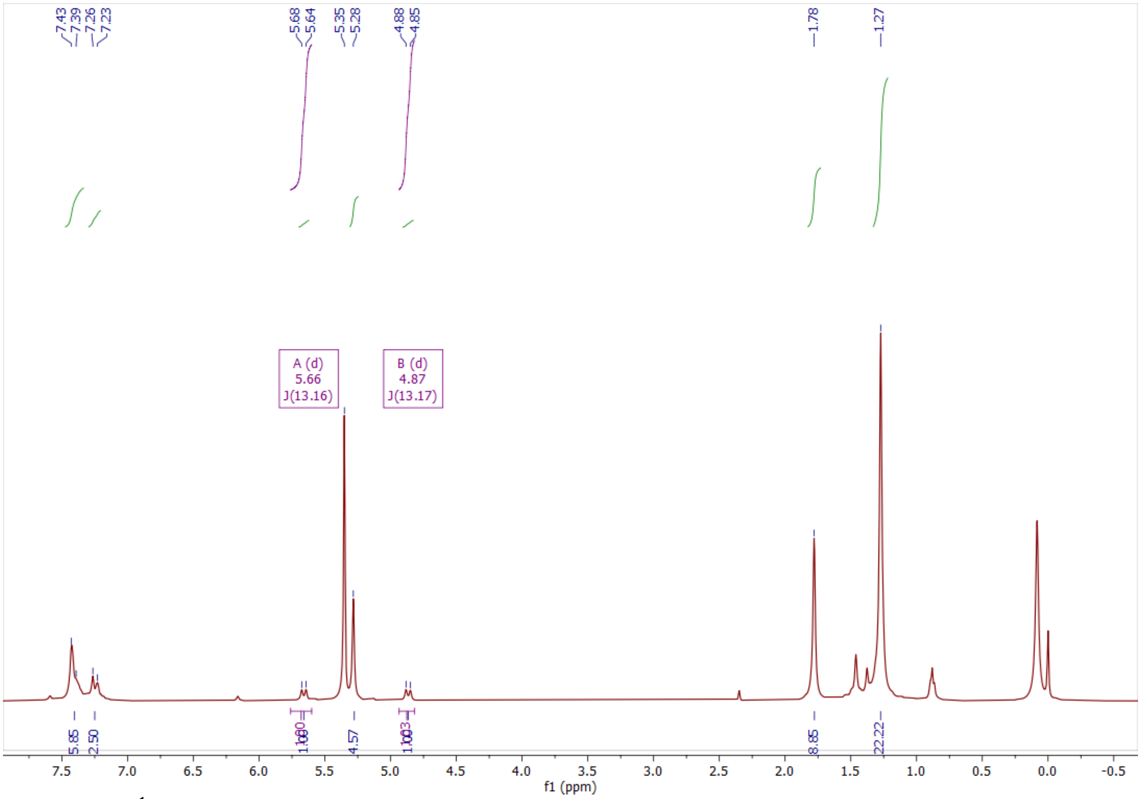

Figure S19. ^{1}H NMR spectrum of compound **3b.1** (CD_2Cl_2, 253 K).

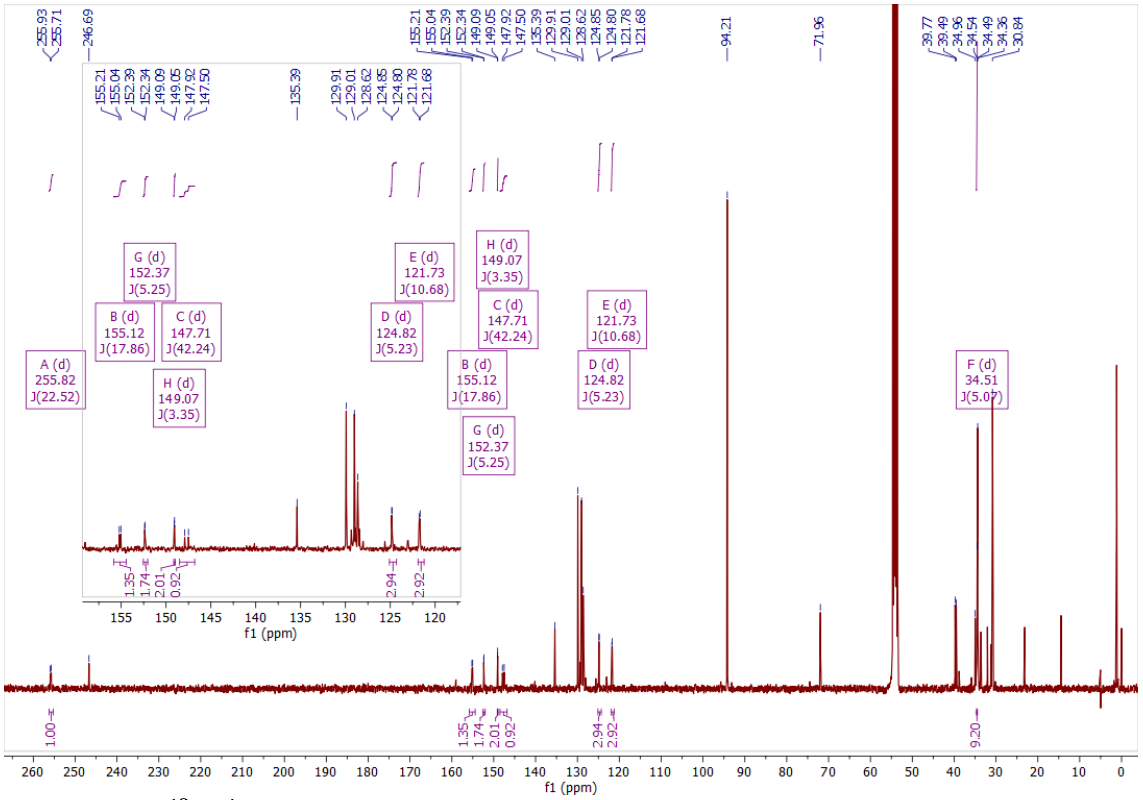

Figure S20. $^{13}C\{^{1}H\}$ NMR spectrum of compound **3b.1** (CD_2Cl_2, 253 K).

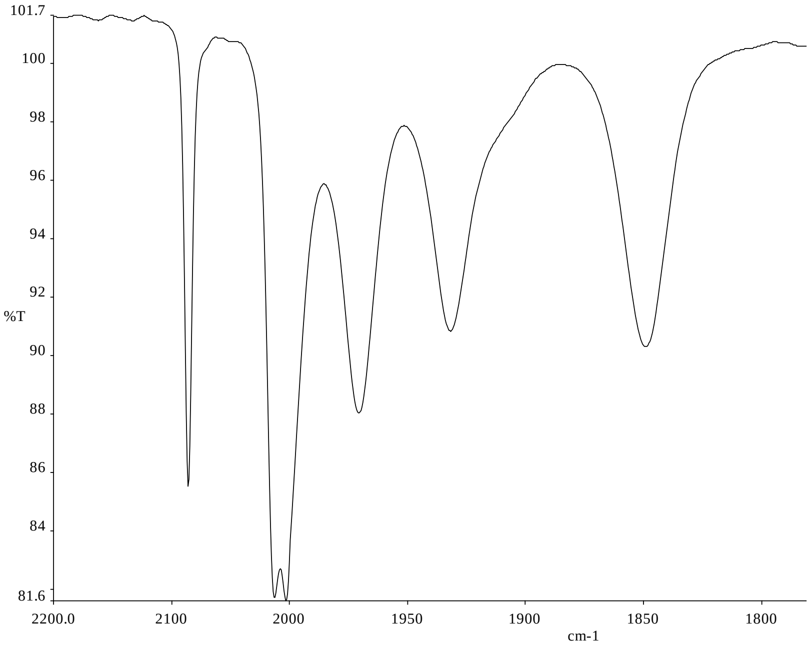

Figure S21. IR spectrum of compound **3b.2** in dichloromethane solution.

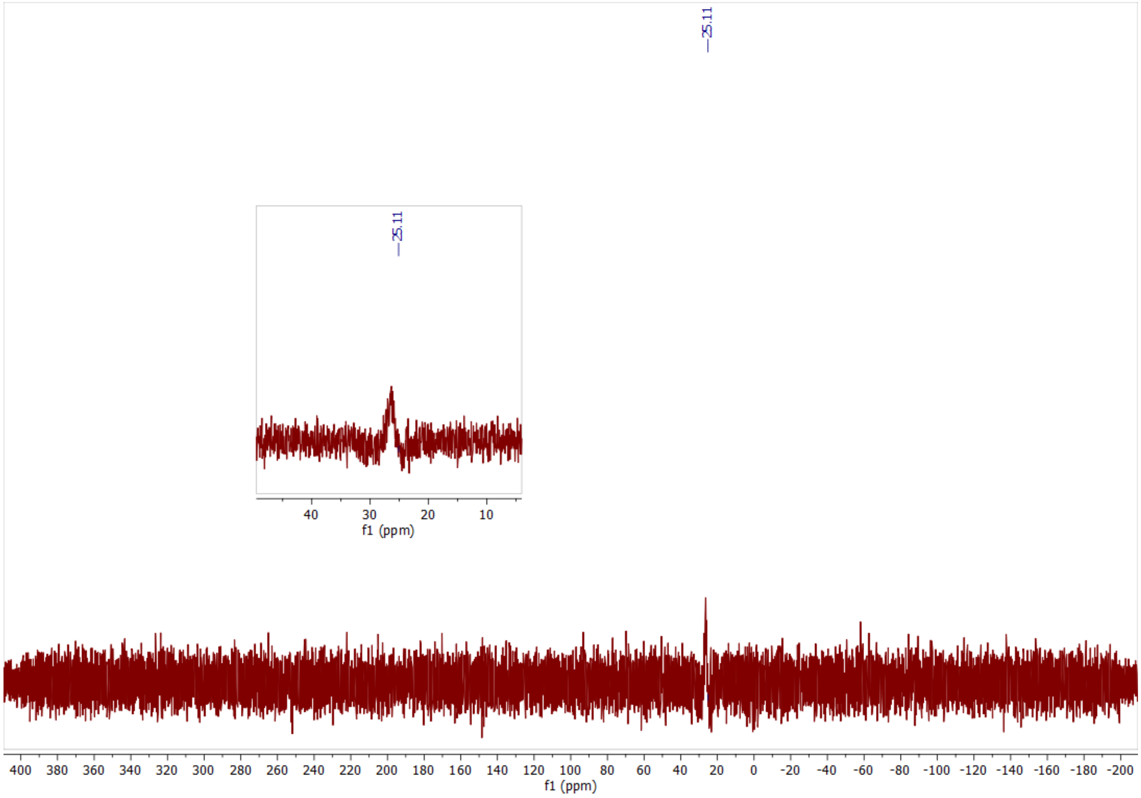

Figure S22. $^{31}P\{^{1}H\}$ NMR spectrum of compound **3b.2** (CD_2Cl_2).

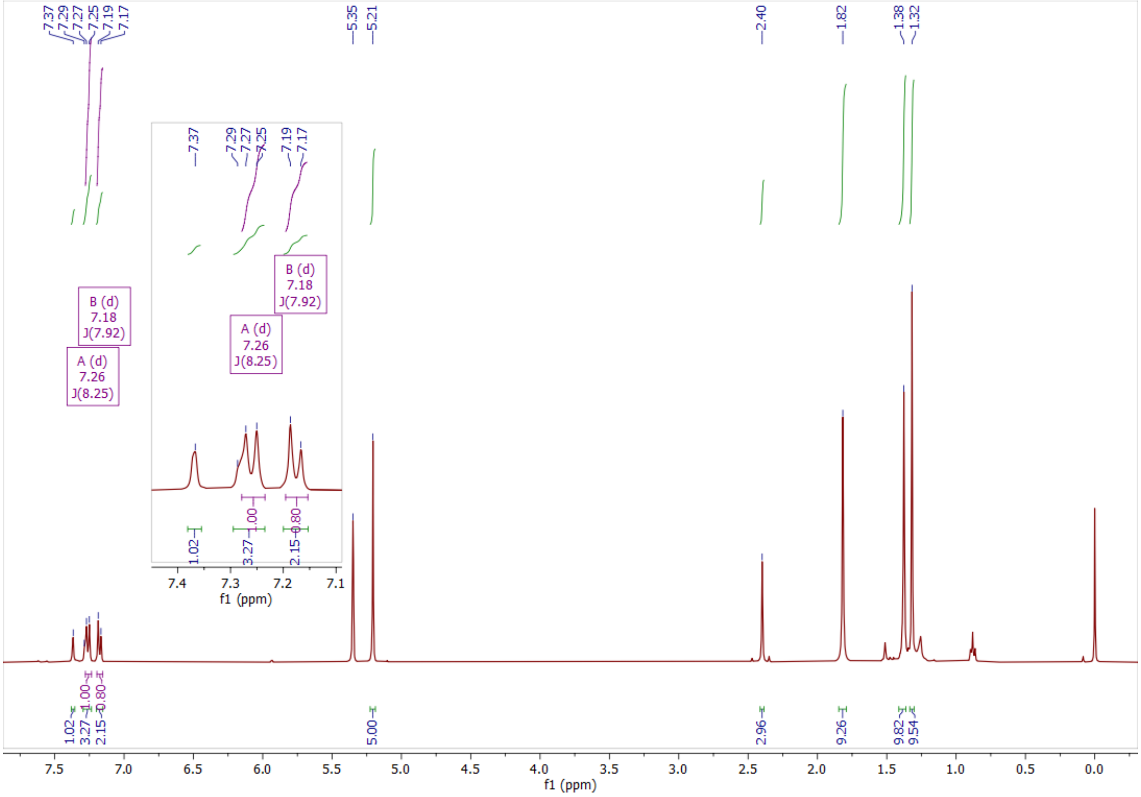

Figure S23. ^{1}H NMR spectrum of compound **3b.2** (CD_2Cl_2, 253 K).

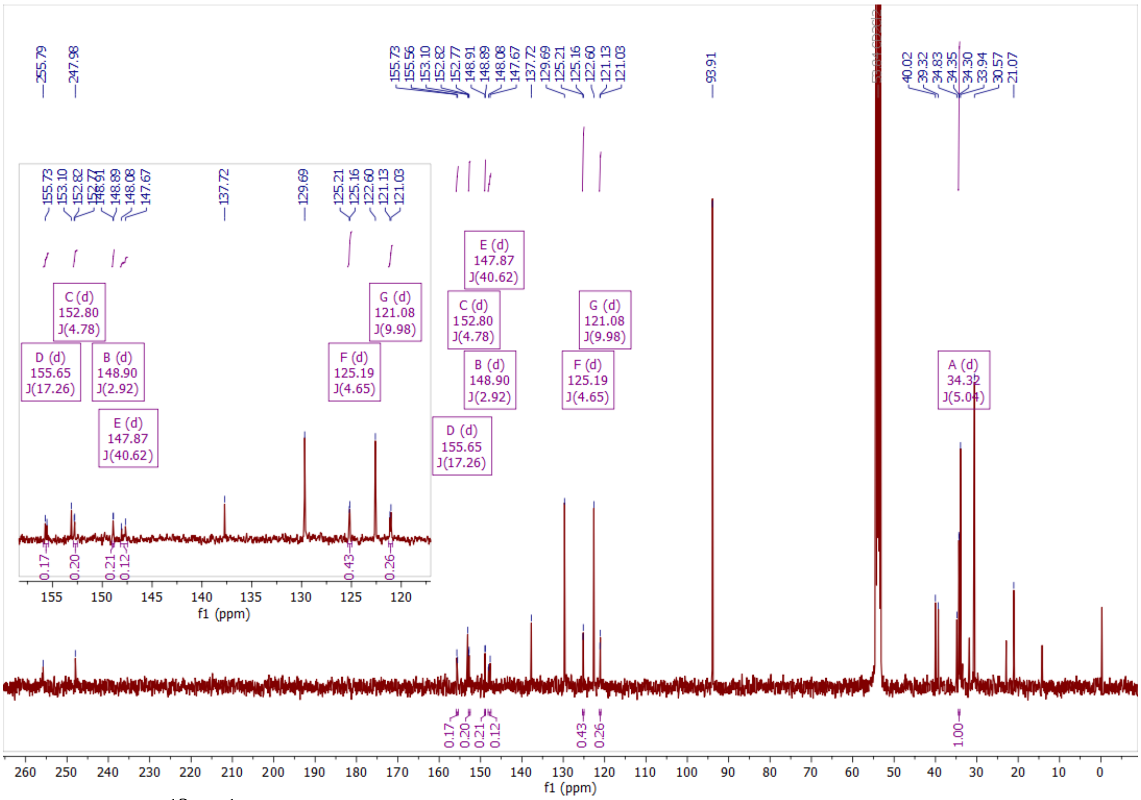

Figure S24. $^{13}C\{^{1}H\}$ NMR spectrum of compound **3b.2**(CD_2Cl_2, 253 K).

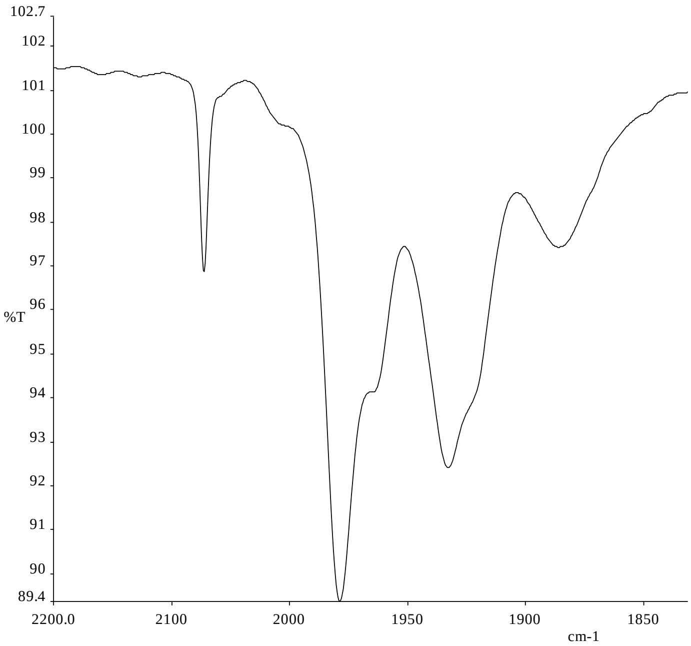

Figure S25. IR spectrum of compound **4a** in dichloromethane solution.

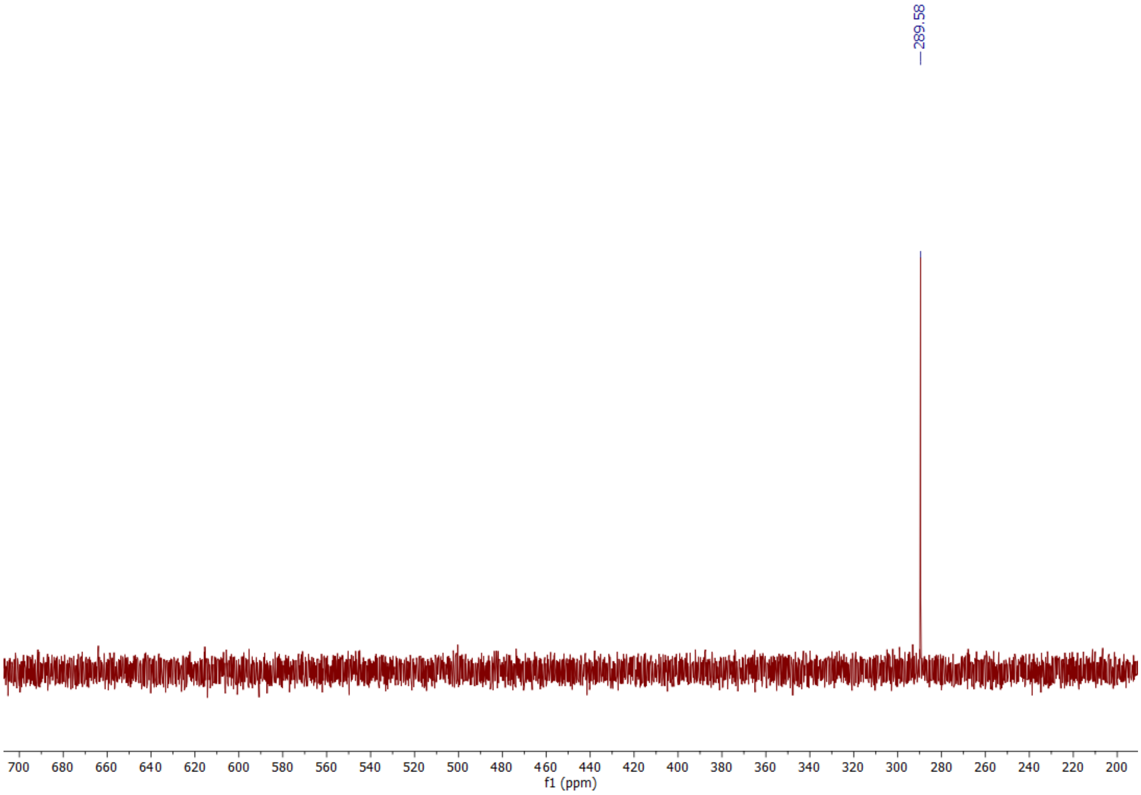

Figure S26. $^{31}P\{^{1}H\}$ NMR spectrum of compound **4a** (CD_2Cl_2).

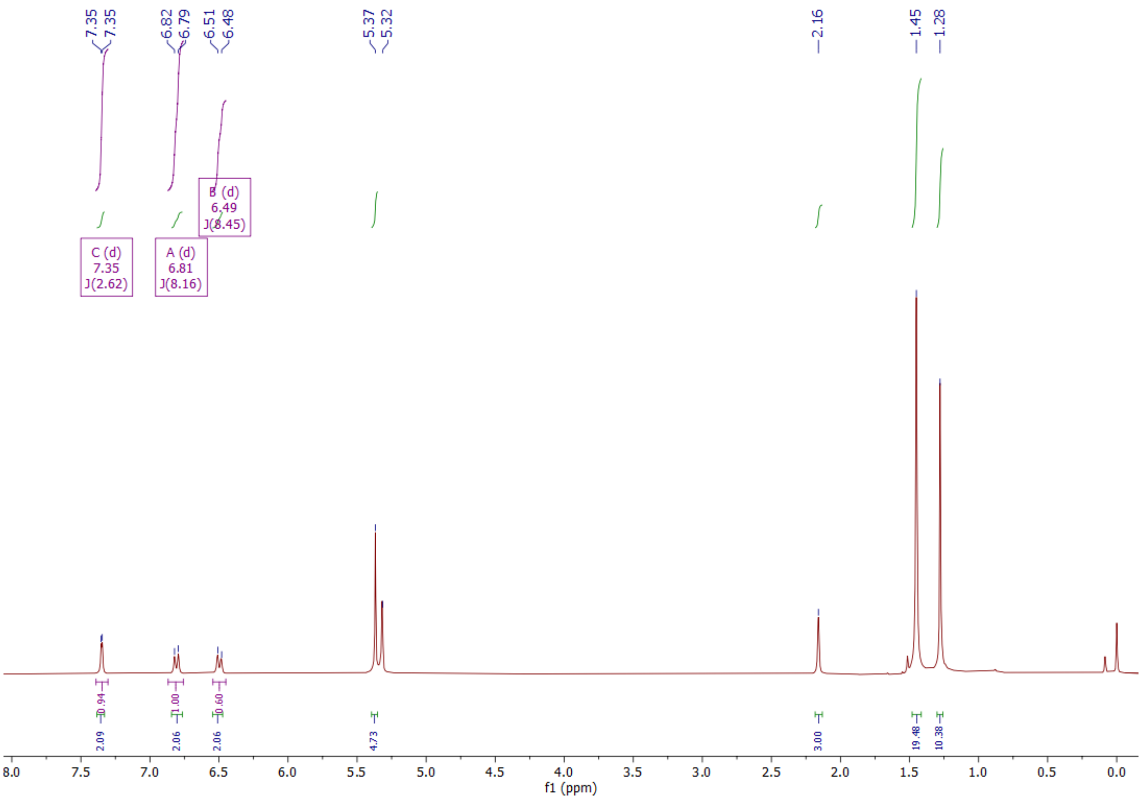

Figure S27. ^{1}H NMR spectrum of compound **4a** (CD_2Cl_2).

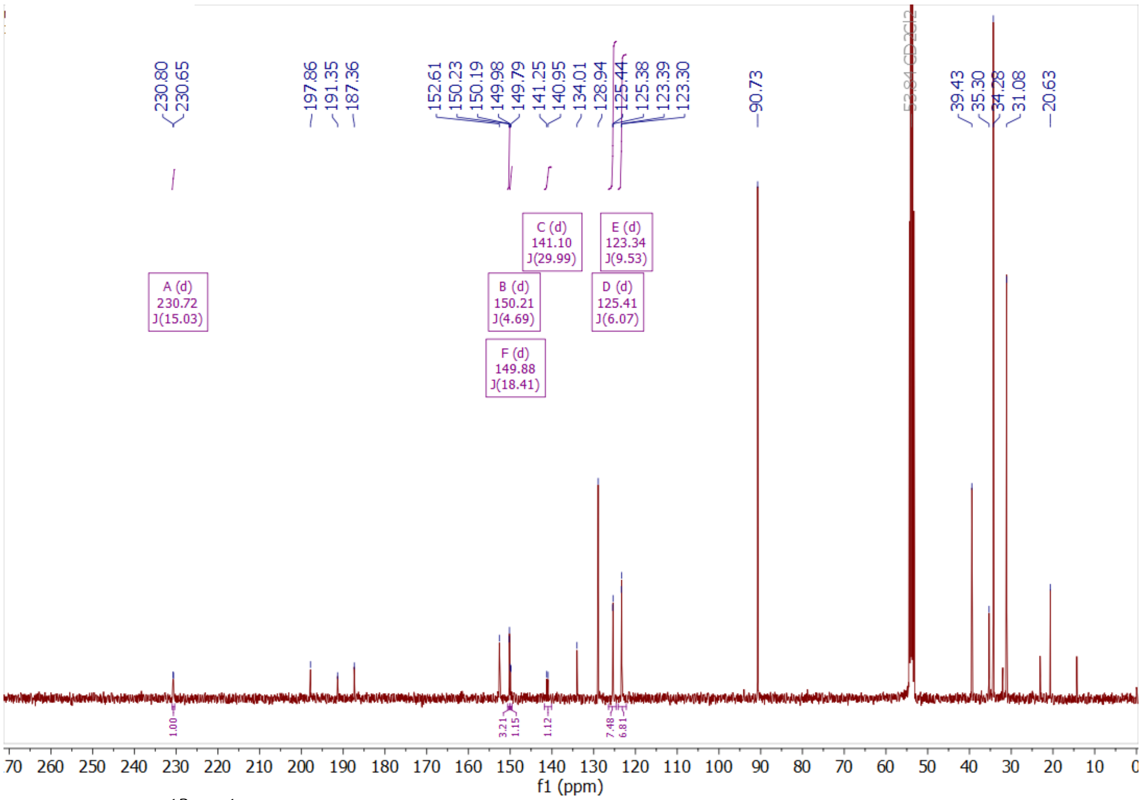

Figure S28. ^{13}C{^{1}H} NMR spectrum of compound **4a** (CD_2Cl_2).

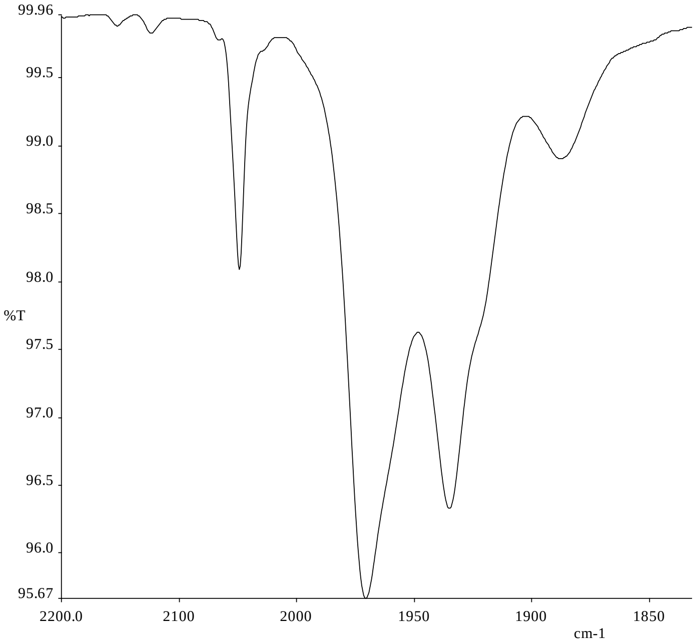

Figure S29. IR spectrum of compound **4b** in dichloromethane solution.

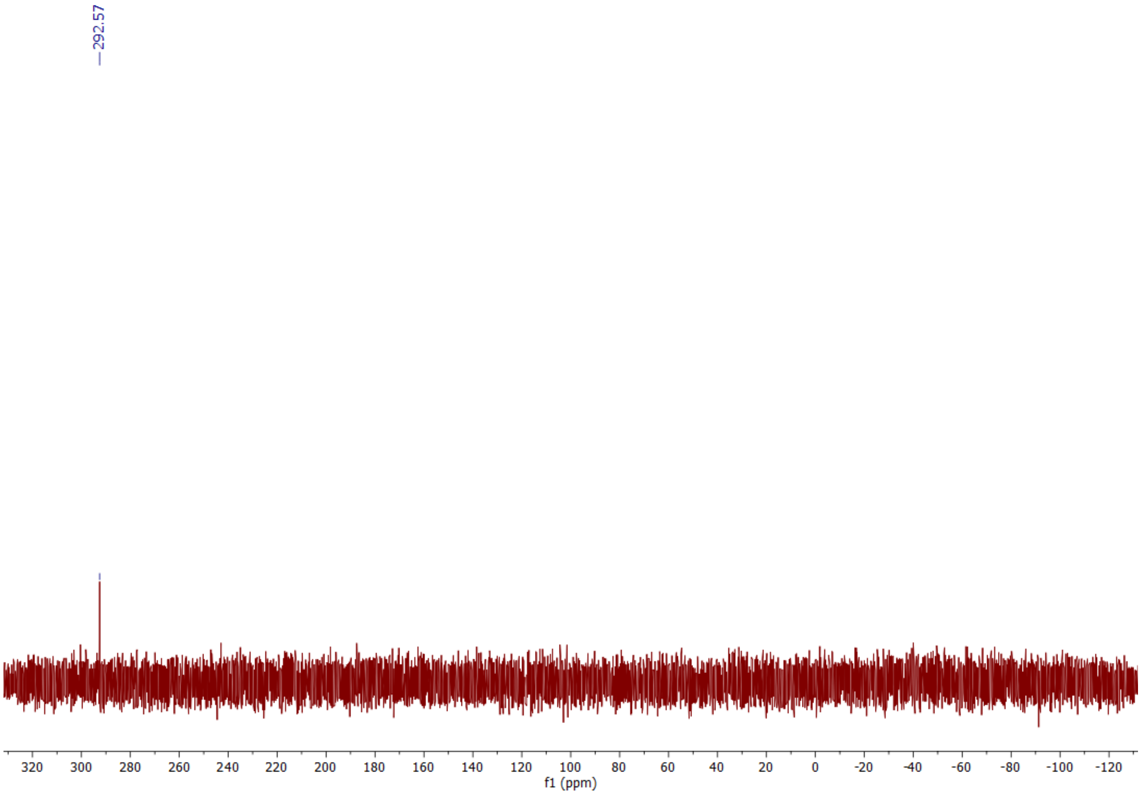

Figure S30. $^{31}P\{^{1}H\}$ NMR spectrum of compound **4b** (CD_2Cl_2).

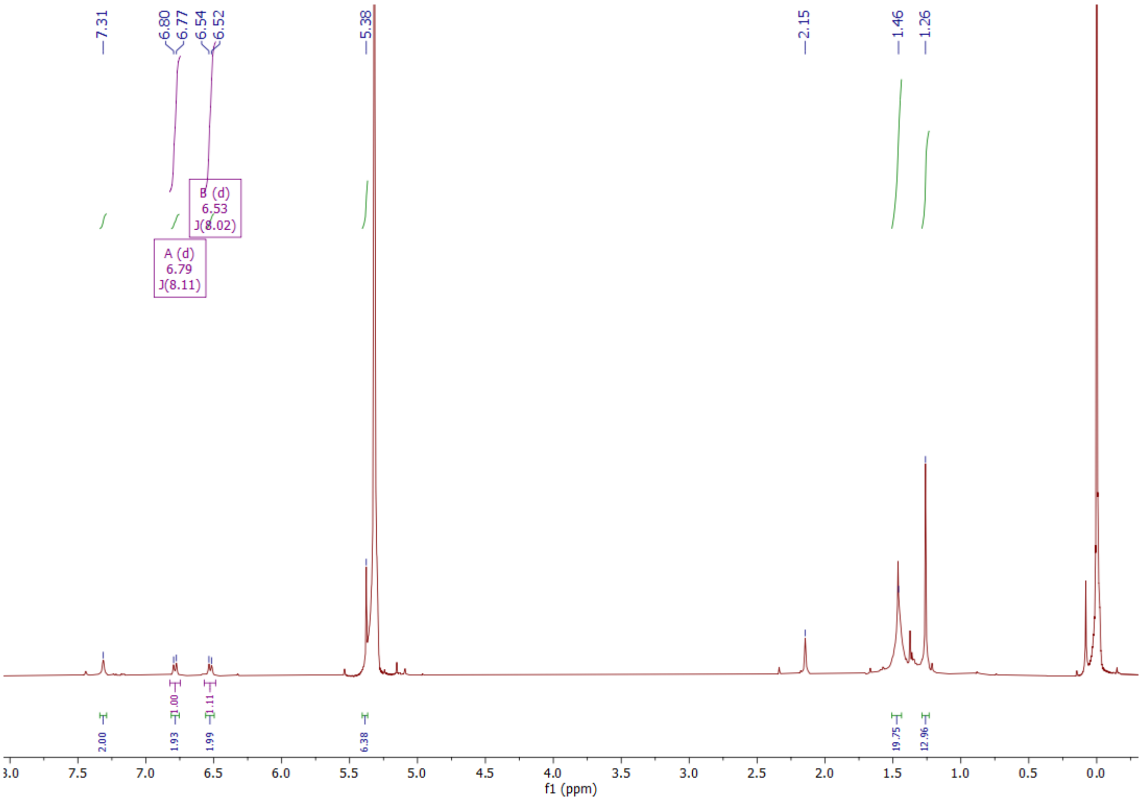

Figure S31. ^{1}H NMR spectrum of compound **4b** (CD_2Cl_2).

.

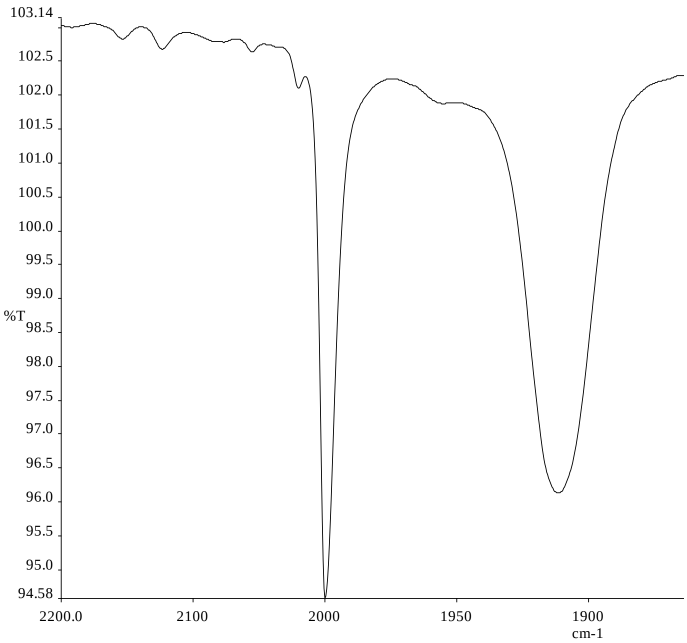

Figure S32. IR spectrum of compound **5.1** in dichloromethane solution.

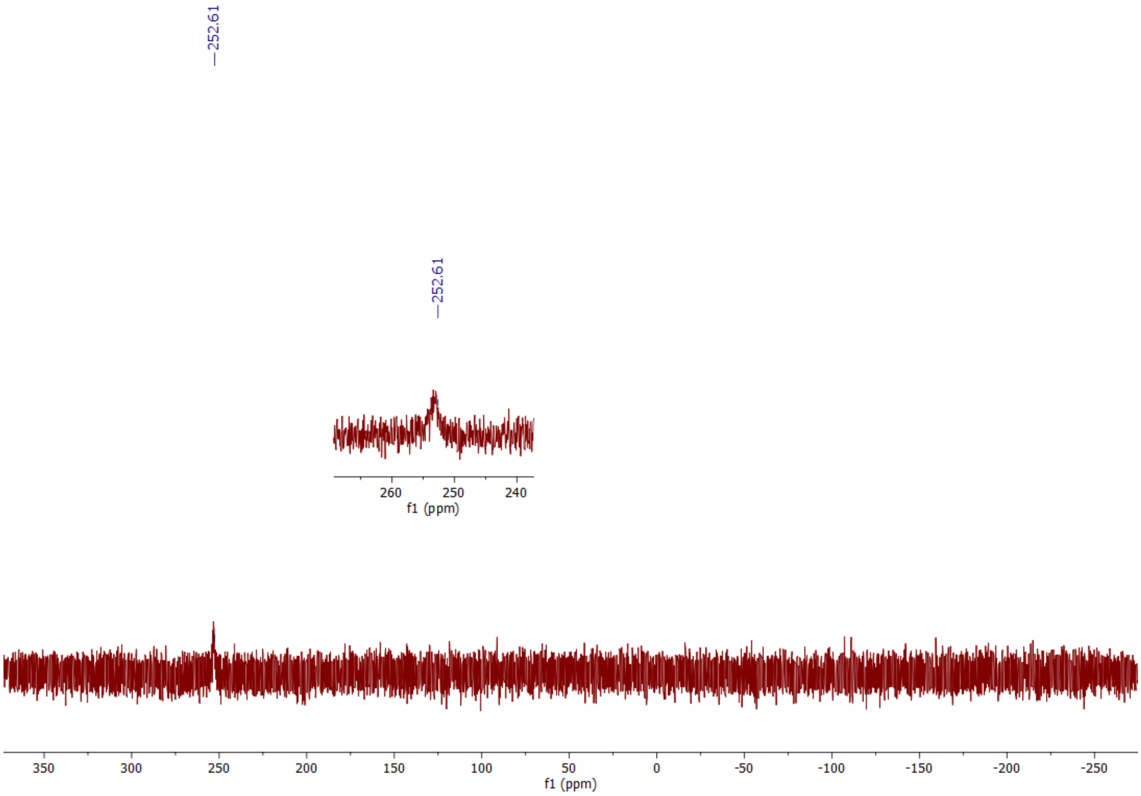

Figure S33. $^{31}P\{^{1}H\}$ NMR spectrum of compound **5.1** (CD_2Cl_2).

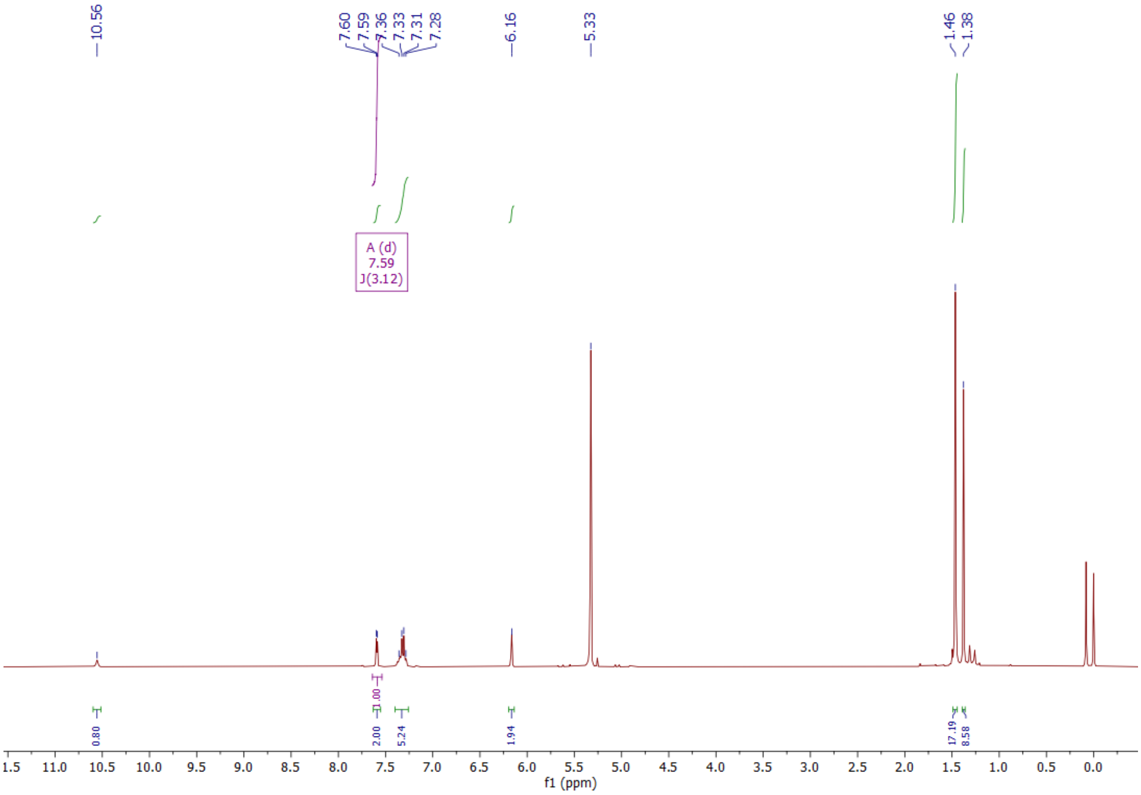

Figure S34. ^{1}H NMR spectrum of compound **5.1** (CD_2Cl_2).

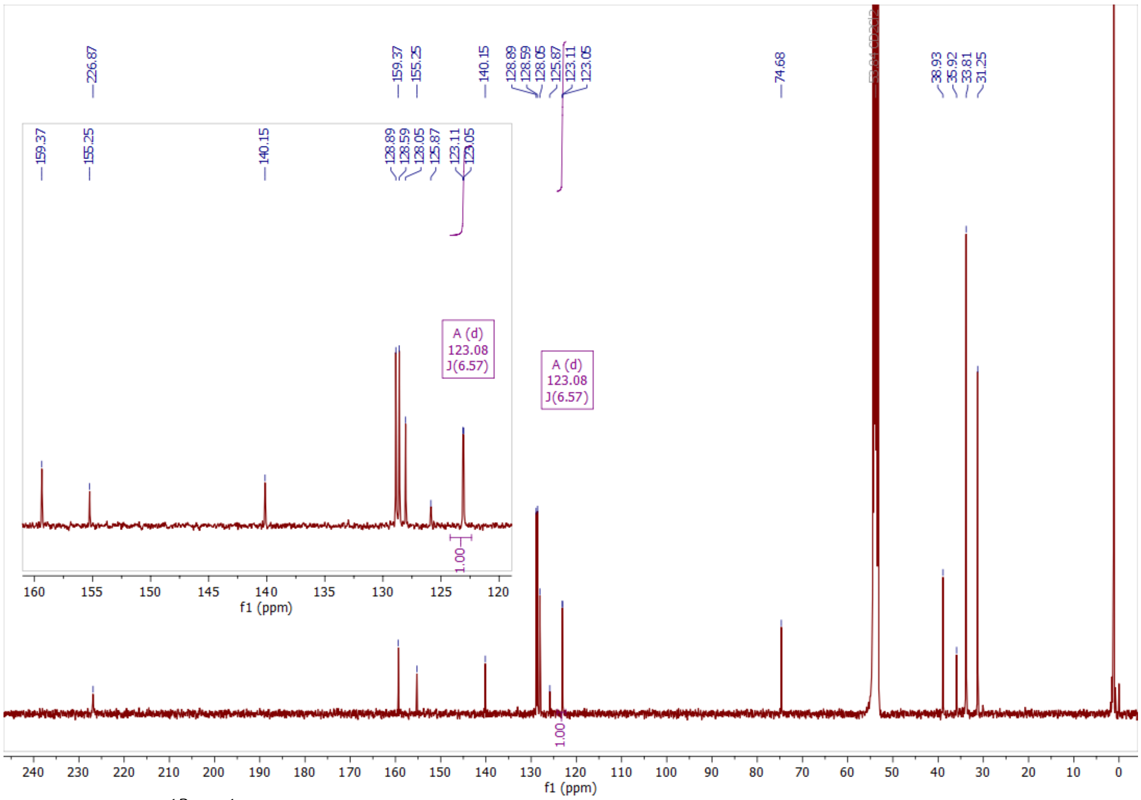

Figure S35. $^{13}C\{^{1}H\}$ NMR spectrum of compound **5.1** (CD_2Cl_2).

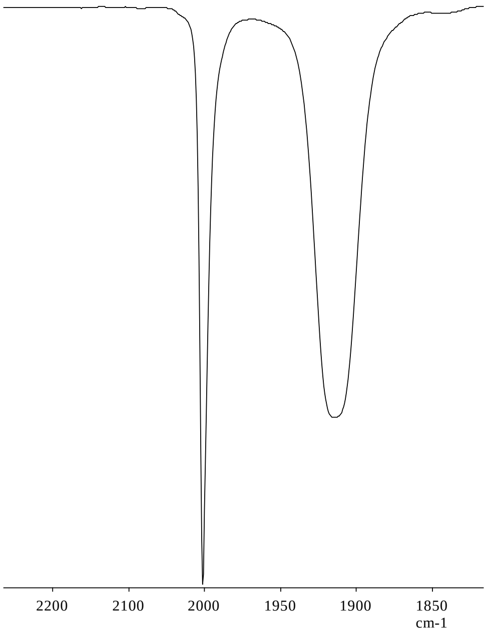

Figure S36. IR spectrum of compound **5.2** in dichloromethane solution.

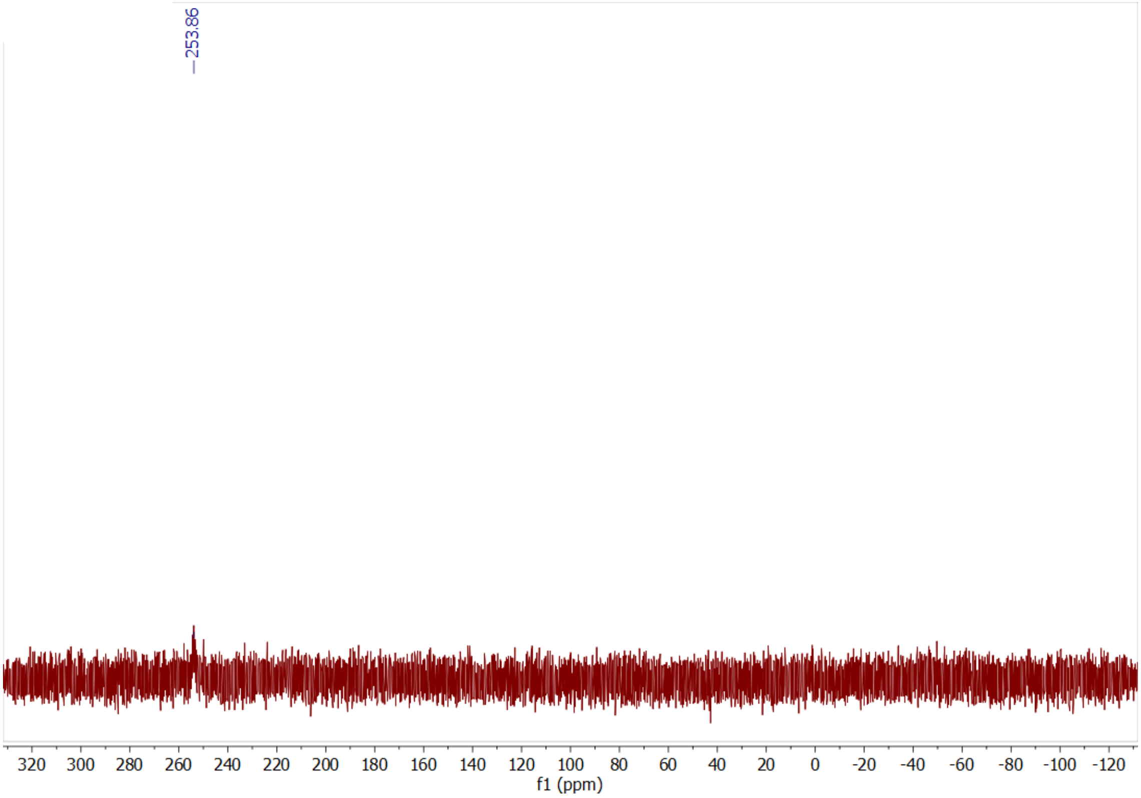

Figure S37. $^{31}P\{^1H\}$ NMR spectrum of compound **5.2** (CD_2Cl_2).

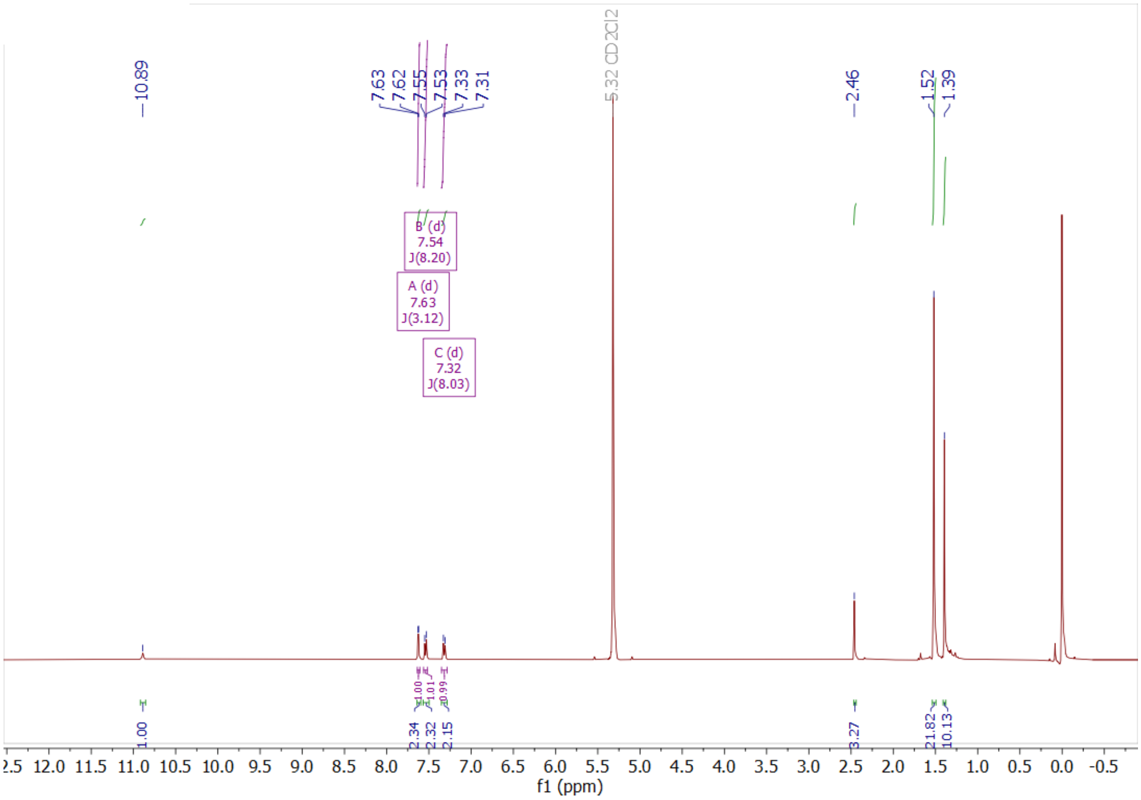

Figure S38. ^{1}H NMR spectrum of compound **5.2** (CD_2Cl_2).

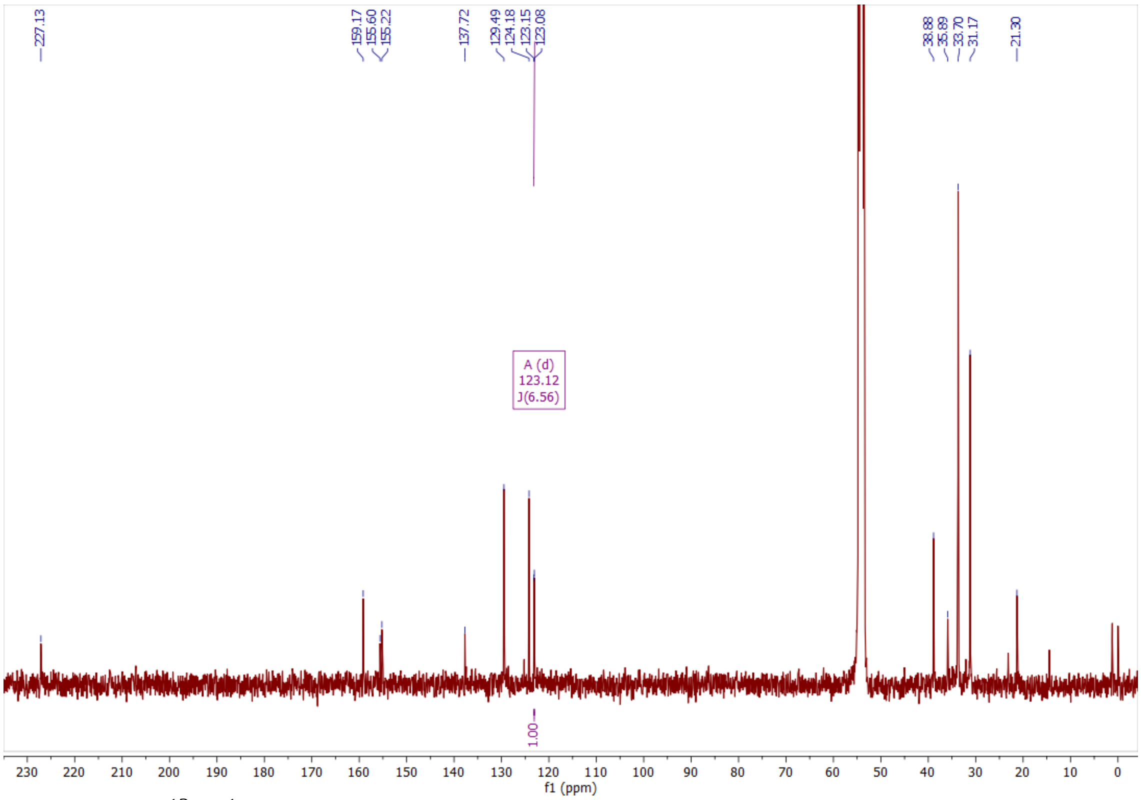

Figure S39. $^{13}C\{^{1}H\}$ NMR spectrum of compound **5.2** (CD_2Cl_2).
